# Integrating belowground recovery into tropical forest restoration design and monitoring

**DOI:** 10.1093/biosci/biaf097

**Published:** 2025-07-14

**Authors:** Laura Toro, Leland K Werden, Shalom D Addo-Danso, Kelly M Andersen, Sarah Batterman, Matilde M Bragadini, Pooja Choksi, Rebecca J Cole, Liza S Comita, Daniela Cusack, Daisy H Dent, Lee H Dietterich, Joshua B Fisher, Katrin Fleischer, Lucia Fuchslueger, Nohemi Huanca-Nunez, Janey R Lienau, Lindsay A McCulloch, Ember M Morrissey, Jennifer S Powers, Mareli Sánchez-Juliá, Oscar Valverde-Barrantes, Anita Weissflog, Michelle Y Wong

**Affiliations:** Center for Conservation and Sustainable Development, Missouri Botanical Garden, St. Louis, Missouri, Yale Center for Natural Carbon Capture, School of the Environment, Yale University, New Haven, Connecticut, United States; ETH Zurich, Zurich, Switzerland; Forest and Climate Change Division of the Forestry Research Institute of Ghana, Fumesua, Ghana; Latin America Division of the Missouri Botanical Garden, St. Louis, Missouri, Asian School of the Environment, Nanyang Technological University, Singapore; Cary Institute of Ecosystem Studies, Millbrook, New York, United States; ETH Zurich, Zurich, Switzerland; Department of Forest Resources, University of Minnesota Twin Cities, St. Paul, Minnesota, United States; ETH Zurich, Zurich, Switzerland; School of the Environment, Yale Center for Natural Carbon Capture, Yale University, New Haven, Connecticut, United States; Smithsonian Tropical Research Institute, Gamboa, Panama; Department of Ecosystem Science and Sustainability, Colorado State University, Fort Collins, Colorado, United States; Smithsonian Tropical Research Institute, Gamboa, Panama; ETH Zurich, Zurich, Switzerland; Smithsonian Tropical Research Institute, Gamboa, Panama; Max Planck Institute of Animal Behavior, Konstanz, Germany; Department of Ecosystem Science and Sustainability, Colorado State University, Fort Collins, Colorado, and with Haverford College, Haverford, Pennsylvania, United States; Chapman University System, Orange, California, United States; Vrije Universiteit Amsterdam, Amsterdam, Netherlands; University of Vienna, Vienna, Austria; School of the Environment, Yale Institute for Biospheric Studies, Yale University, New Haven, Connecticut, United States; School of the Environment, Yale University, New Haven, Connecticut, United States; University of South Florida, Tampa, Florida, United States; Smithsonian Tropical Research Institute, Gamboa, Panama; Department of Biology, West Virginia University, Morgantown, West Virginia, United States; Department of Plant Biology, University of Minnesota Twin Cities, St. Paul, Minnesota, United States; Department of Ecology and Evolutionary Biology, Yale University, New Haven, Connecticut, United States; Florida International University, Miami, Florida, United States; School of the Environment, Yale Institute for Biospheric Studies, Yale University, New Haven, Connecticut, United States; Department of Ecology and Evolutionary Biology, Yale University, New Haven, Connecticut, United States; Smithsonian Tropical Research Institute, Gamboa, Panama

**Keywords:** ecosystem recovery, edaphic properties, indicators, monitoring frameworks, soil

## Abstract

There is growing recognition that tropical forest restoration is key for sequestering carbon and enhancing ecosystem resilience. Soils, roots, and soil biota are central to ecosystem function and services, but belowground recovery is largely overlooked in restoration monitoring frameworks. Here, we outline current understanding of the links between above- and belowground recovery in tropical forests by examining how belowground properties before and after intervention influence recovery; by evaluating whether aboveground recovery can serve as a proxy for belowground dynamics; and by proposing a blueprint for monitoring dynamic soil physical (bulk density, aggregate stability), chemical (organic matter or carbon, pH), and biological properties (decomposition rate, macrofauna abundance) in resource-constrained projects. Although we highlight some aboveground proxies for assessing belowground recovery, a better understanding of relationships between above- and belowground indicators across diverse restoration interventions remains essential. Overall, we provide an actionable path toward integrating belowground recovery into restoration design and assessment.

Natural climate solutions have emerged as key levers in addressing the climate crisis, because an estimated one-third of anthropogenic emissions could be mitigated by preserving, improving the management of, and restoring anthropogenically disturbed terrestrial ecosystems (Griscom et al. 2017, Buma et al. 2024). Numerous studies have emphasized the potential to increase carbon stored in forests globally if sustainable management and ecosystem restoration are implemented effectively and at scale (less than 200 petagrams of carbon total; Bastin et al. 2019, Walker et al. 2022, Mo et al. 2023). *Ecological restoration* is the process of assisting the recovery of an ecosystem that has been degraded, damaged, or destroyed (SER 2002). Restoring previously forested areas, particularly in the tropics, can play a valuable role in not only drawing down carbon but also in conserving biodiversity and promoting ecosystem recovery (Busch et al. 2019). This realization, among others, has catalyzed the establishment of global restoration initiatives (e.g., the Bonn Challenge; www.bonnchallenge.org) employing a gradient of interventions from natural regeneration to tree planting (i.e., assisted restoration *sensu* Chazdon et al. 2021) across the tropics (Holl 2017).

To thoroughly assess the effectiveness of forest restoration efforts on the ground, restoration actors need comprehensive and standardized monitoring frameworks (Gatica-Saavedra et al. 2017, Giles et al. 2024). Recent efforts have led to a suite of restoration indicator frameworks (e.g., Gann et al. 2022, UN Decade on Ecosystem Restoration 2024), visual tools (the Recovery Wheel; Gann et al. 2019), and data platforms (e.g., Restor-Crowther et al. 2022, IUCN Restoration Barometer 2022) to assess project outcomes and build on commonly measured aboveground vegetation indicators, such as plant growth, survival, richness, and canopy cover, and plant–animal interactions such as pollination (Gatica-Saavedra et al. 2017). Belowground components, including soils, roots, and soil biota, play essential roles in maintaining key ecological functions across diverse ecosystems (Bardgett and van der Putten 2014, Adhikari and Hartemink 2016, van der Sande et al. 2023). However, the recovery of belowground properties remains largely unmeasured in restoration projects (Mendes et al. 2019, Allek et al. 2023, Gatica-Saavedra et al. 2023, Duque et al. 2025). This is particularly notable given the critical role of the recovery of belowground interactions, both mutualistic and antagonistic, in shaping forest diversity and productivity (Kardol and Wardle 2010). For example, across the Latin American tropics, restoration practitioners indicated that they typically only evaluate aboveground recovery, despite having broad goals of recovering ecosystem processes and interactions (Cole et al. 2024). This gap is prevalent not only among individual projects but also within global restoration monitoring frameworks. For instance, a global stocktaking exercise that generated more than 4500 indicators of restoration success included only one belowground recovery indicator (soil carbon) out of the 61 final indicators suggested for prioritization (Gann et al. 2022). It may be possible to derive recovery of belowground properties using aboveground proxies; for example, community-level plant leaf nutrient content can be correlated with soil fertility across environmental gradients (Reich 2014). However, few studies have explicitly investigated links between above- and belowground recovery over time to determine whether these proxies are reliable in a variety of contexts (but see Bieluczyk et al. 2023, who linked the recovery of above- and belowground carbon stocks, and Dorrough et al. 2023 who found that soil organic matter recovery was coupled with the recovery of forest structure).

The general lack of data on belowground recovery hampers our ability to understand ecosystem recovery dynamics. Few tropical forest restoration efforts collect belowground indicators before and after intervention, precluding our ability to 1) tailor interventions on the basis of the state of belowground properties at a site, 2) to evaluate the suitability of the interventions applied within a specific context, 3) to track trajectories of belowground recovery and understand appropriate timescales for monitoring, and 4) to understand how above- and belowground recovery processes modulate each other (Callaham et al. 2008, Mendes et al. 2019, Farrell et al. 2020). In forests, the loss of vegetation cover can heavily affect a suite of belowground processes, such as water and nutrient cycling and carbon sequestration, through the soil profile (van Straaten et al. 2015, Veldkamp et al. 2020). Therefore, understanding the initial state and recovery rates of belowground processes is key to the success of restoration outcomes aboveground, because soils provide nutrients and water and serve as the substrate for key symbionts that plants need to successfully recolonize degraded areas (Deyn and Kooistra 2021). This highlights a critical need to better integrate belowground processes into restoration practice and assessment and to understand how the recovery of above- and belowground processes is linked so that projects can be adaptively managed on the basis of observed recovery trajectories (Kardol and Wardle 2010).

Characterizing the capacity of soils to sustain plants, animals, and humans—often referred to as soil health (USDA-NRCS 2025)—before and after intervention provides valuable information on potential barriers to recovery, as well as drivers of restoration outcomes (Hatten and Liles 2019, Nolan et al. 2021, UNCCD 2022). For example, the speed of belowground recovery can vary by orders of magnitude across sites because of prior land use (Poorter et al. 2021) and soil fertility (van der Sande et al. 2023). Although soil nitrogen can rebound within a few decades, important nonrenewable soil base cations and phosphate susceptible to soil weathering and erosion may not recover as a direct result of restoration interventions (Lambers et al. 2008, Swinfield et al. 2020). Soil phosphorus availability in particular is highly context dependent, determined by the predisturbance availability and other soil properties, and highly susceptible to land-use changes (Bauters et al. 2021, Swinfield et al. 2020, van der Sande et al. 2023). Not considering nutrient availability or adding specific amendments (e.g., during species selection for an assisted restoration project) can impede aboveground recovery and hamper progress toward restoration goals (Aide and Cavelier 1994, Mendes et al. 2019, Nolan et al. 2021).

In the present article, we outline current understanding of the linkages between above- and belowground recovery in tropical forest restoration. To do so, we describe how the state of belowground properties before and after restoration intervention influences ecosystem recovery trajectories, discuss which aspects of belowground recovery can potentially be inferred from aboveground recovery, and provide pragmatic guidelines for how existing and emerging monitoring tools can be leveraged to track belowground recovery of soil physical, chemical, and biological properties, given the tight budgets and personnel limitations of most tropical forest restoration projects. Overall, we present a blueprint for holistic restoration design and tropical forest recovery assessment, aiming to identify and implement pragmatic solutions to address key knowledge gaps in belowground recovery. Throughout, we define recovery as progress toward clearly defined ecological or functional goals, recognizing that these goals vary widely across projects depending on ecological, social, and climatic contexts (Gann et al. 2019).

## Belowground properties influence recovery: An integral part of each stage of the restoration process

**Figure 1.**
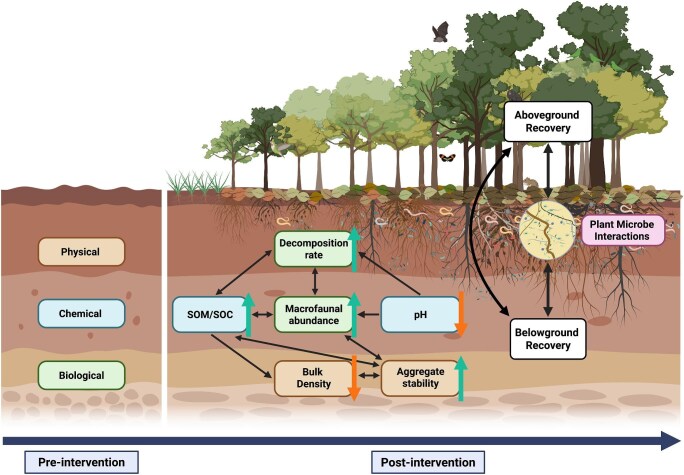
Conceptual diagram illustrating the importance of considering the state of soil properties preintervention in tropical forest restoration projects; these influence the trajectory of both above- and belowground recovery. To establish a baseline preintervention and monitor recovery postintervention, we suggest six key indicators covering dynamic soil physical (bulk density, aggregate stability), chemical (soil organic matter/soil organic carbon, pH), and biological (decomposition rate, macrofaunal abundance) properties. The black arrows represent known links among soil indicators and between soil properties and above- and belowground recovery processes. The color arrows indicate the expected direction of change following restoration: green arrows for increases and orange arrows for decreases (Veldkamp et al. 2020, Van der Sande et al. 2023). Recovery is also shaped by the reestablishment of plant–microbe interactions, which mediate feedback loops between vegetation and soil processes.

**Pre-intervention—**Land degradation due to land-cover change removes native vegetation, restructures animal and microbial communities, and can alter the physical, chemical, and biological properties of soils, limiting native plant establishment and modifying ecosystem function (Olsson et al. 2019). These changes can shape the trajectory and success of restoration because soil conditions are one key factor limiting the recovery of native vegetation (Veldkamp et al. 2020). Recovery trajectories depend heavily on prior land use and the degree of soil degradation (Bonner et al. 2019, Bauters et al. 2021). Assessing whether to monitor soil recovery over time can be guided by observed declines in soil properties following land-cover change relative to a more intact reference site. Therefore, understanding how the initial state of physical, chemical, and biological soil properties influence overall recovery rates is key to implementing effective restoration interventions, defining restoration goals, and tracking the recovery of these properties (figure 1).

In terms of soil physical properties, the conversion of forests to other land uses often modifies water infiltration, retention, and redistribution, negatively affecting the recovery rates of native forest cover (Meli et al. 2024). Land-use changes primarily affect soil structure, reducing soil aggregation and porosity, and increasing bulk density. Converting forests to agriculture can also reduce soil infiltration capacity (i.e., decreased porosity) because of factors such as reduced soil faunal activity or a loss of water holding capacity due to decreased organic matter content. These changes can increase surface runoff, soil loss, and erosion (Giertz et al. 2005). Moreover, soil compaction from grazing animals (e.g., cattle) or machinery in tropical forests can increase bulk density, leading to reduced infiltration rates, limited root growth, and decreased water storage.

High soil bulk density following soil compaction limits seedling establishment because roots cannot penetrate the soil and access nutrients or water, constraining vegetation recovery (e.g., Hattori et al. 2013). However, over decades soil bulk density can often recover (i.e., decrease) under natural regeneration (van der Sande et al. 2023) and assisted restoration (Mendoza-Vega et al. 2020). Therefore, understanding the initial state of soil physical properties at a given site can help determine how to tailor restoration interventions to optimize recovery, evaluate project outcomes, and estimate how long it will take the system to recover.

The state of soil chemical properties before and after disturbance also dictates the path of recovery trajectories. Research across 21 Neotropical forest chronosequences—encompassing a disturbance gradient from active agriculture to regenerating forests of varying ages, and old-growth forests—showed that both the magnitude and direction of changes in soil carbon and nitrogen depended strongly on local conditions such as soil type and land-use history (van der Sande et al. 2023). For example, deforestation on fertile soils led to declines in soil carbon stocks, likely because of intensive land use, followed by strong recovery during forest succession. In contrast, on less fertile soils, carbon stocks did not change after disturbance or during recovery, possibly because of lower overall plant productivity and limited fine root and litter inputs, which reduce both disturbance-driven losses and recovery potential. Although the nitrogen cycle can recover substantially during natural regeneration (Figueiredo et al. 2019), it is not clear how complex land-use legacies and landscape factors affect other biogeochemical cycles, such as the phosphorus cycle in particular, at restored sites (Sullivan et al. 2019, Jakovac et al. 2021). In fact, soil chemical processes (e.g., oxidation reduction, adsorption and desorption) can be so altered by disturbance that the recovery of predisturbance plant communities may not be possible without extremely high fertilizer input that is often not realistic within a restoration context (Soper et al. 2024). As such, understanding soil chemical properties can help to tailor restoration interventions to the context—for example by liming when the pH is lower than 5 or choosing to plant native nitrogen-fixing tree species to rapidly increase soil nitrogen availability (Nichols et al. 2001, Hoogmoed et al. 2014, Lewis et al. 2019).

Following a disturbance, soil biological properties (e.g., microbial diversity, macroinvertebrate richness, nitrogen fixation) also have a major influence on forest recovery during restoration, and some of these properties can take decades to recover (Veldkamp et al. 2020). Microbial communities, including fungi and bacteria, form important relationships with vegetation that help make water and nutrients available to plants and enhance their uptake (Pereira et al. 2022, Leite et al. 2023). A recent global meta-analysis of inoculation experiments found the addition of microorganisms from undisturbed reference habitats to degraded soils increased plant productivity by an average of 64% across various ecosystems (Averill et al. 2022). However, dynamics such as increased nutrient availability following agricultural land use can reduce root colonization by mycorrhizal fungi (e.g., Delavaux et al. 2017). Therefore, it is important to consider how plant–microbe interactions can be reestablished during restoration because these symbioses reduce barriers to the reestablishment of native vegetation (Averill et al. 2022, McCulloch et al. 2024).

In addition, the negative effects of specialist soil pathogens (e.g., Allen et al. 2003) and the positive effects of soil fauna (e.g., Nielsen 2019) tend to become more prevalent beyond the early stages of tropical forest restoration. These pathogens may play a role in promoting shifts in tree species composition and increasing diversity over time. Furthermore, soil fauna such as termites, ants, worms, beetles, and other macrofauna can reduce soil compaction and increase water infiltration, nutrient availability, soil organic matter, and seed dispersal (Magalhães et al. 2018, Benbow et al. 2019, Nielsen 2019, Parkhurst et al. 2021). Microbes and detritivores can also have strong control on the rate of litter decomposition and, therefore, nutrient release and cycling (Hättenschwiler and Gasser 2005, González and Lodge 2017, Stone et al. 2020). Taken together, these studies show that cataloging the initial state of soil biological properties can help determine whether specific assisted interventions are needed to catalyze recovery, in the form of microbial amendments (Neuenkamp et al. 2019, Averill et al. 2022) or the reintroduction of soil fauna, which has been tested at the seedling and plot scales (Contos et al. 2021, Morales-Márquez and Meloni 2022)*.*

**Post-intervention**—Once restoration is initiated, recolonizing communities of plants, animals, and microbes influence belowground recovery trajectories through feedback loops and synergies (figure 1). The reassembly of plant communities influences the recovery of belowground processes such as nitrogen fixation (Cusack et al. 2009), root exudation, and nutrient uptake (Homann et al. 2000) and affects litter quality, quantity, and decomposition rates (Laird-Hopkins et al. 2017, Wallwork et al. 2022). For example, the presence of nitrogen-fixing species can increase litter quality and make nutrients more readily available for other plants and microbes, although this varies by species (Hoogmoed et al. 2014). Plant roots are the interface between the above- and belowground components of ecosystems and can modify physical, chemical, and biological properties following disturbance. Root systems can reduce erosion (Demenois et al. 2017) and enhance soil structure (Bergmann et al. 2016) by increasing aggregate stability and hydraulic function (Ola et al. 2015). They can also increase nutrient, amino acid, and sugar availability that can support a diverse community of pathogens, herbivores, decomposers, and symbionts (Frouz 2024). On the other hand, the recovery of fauna postintervention can affect soil nutrients, soil organic matter, and microbial communities via inputs of fecal matter as well as carcasses and excretory compounds (Benbow et al. 2019). Mammals and birds also disperse soil microbes, including mycorrhizal fungi, over long distances either by digging and disrupting the soil (e.g., pigs, primates) or by consuming fruiting bodies and dispersing spores in fecal matter (Vašutová et al. 2019, Paz et al. 2021). Therefore, understanding how plant and animal communities influence the recovery of belowground properties is critical to determining how above- and belowground recovery trajectories are coupled or decoupled within different restoration contexts. Teasing apart these dynamics could also facilitate the estimation of belowground recovery from aboveground recovery, helping to better allocate the limited resources available for restoration monitoring.

## When can we infer belowground from aboveground recovery to simplify the monitoring process?

There is potential to approximate belowground recovery in forest restoration from aboveground data being gathered in existing monitoring efforts. Many tropical forest restoration projects track the recovery of vegetation and are primarily focused on tree biomass or carbon and aspects of vegetation structure (e.g., canopy height and cover; Robinson et al. 2015, Gavito et al. 2021). Less commonly, projects measure vegetation dynamics (e.g., net primary productivity, annual growth rates; Campo and Vázquez-Yanes 2004, Jones et al. 2019) or plant community diversity and composition (e.g., species richness, functional diversity; Evangelista de Oliveira et al. 2021, Cole et al. 2024). These aboveground dynamics provide a window into the recovery of some aspects of belowground physical, chemical, and biological properties that may preclude the need for additional time-intensive data collection by practitioners. However, a Web of Science search and assessment of the literature (*n* = 196 papers total; see supplemental table S1 for the search terms) only revealed 28 papers in which above- and belowground recovery were directly compared in tropical forests. The general lack of studies addressing this topic is not surprising, given that above- and belowground metrics are measured at different temporal and spatial scales and that studying these interactions is complex (van der Putten et al. 2009). Our results highlight significant gaps in our understanding of how recovery of above- and belowground properties are either coupled or uncoupled (table 1).

**Table 1.** Number of studies and relationships between aboveground indicators of recovery (e.g., plant carbon/biomass) and belowground physical, chemical, and biological properties during tropical forest revegetation. The relationships (+, positive; −, negative; ns, non-significant) were extracted from literature found in a systematic search of Web of Science conducted on 31 July 2024 (see supplemental table S1 for search terms and literature list).

Type of property	Belowground metrics	Aboveground metrics						
		Aboveground biomass or carbon	Structure (e.g., height, canopy cover)	Plant diversity	Dynamics (e.g., net primary productivity, tree growth rates)	Tree Composition (e.g., the percentage of old growth species, functional groups or traits)	Faunal communities or function	Leaf litter stocks or depth
Physical	Bulk density	2–^a,b^	2 ns^r,s^	1–^v^		1 ns${_{.}^{\rm s}}$		
	Aggregate stability							
	Soil moisture	2+^c,d^, 1 ns^c^	1+^d^	1+^d^		1+^ac^, –1^ac^		
	Hydraulic conductivity and infiltration rate		+ 1^r^					
	Temperature	1–^c^		1–^a^				1–^a^
Chemical	Organic and total carbon	3+ ^e,f,g^, 4 ns^h,i,j,k^	1 ns^t^	1+^t^	1 ns^k^	1+^ad^		1+^f^, 1 ns${_{.}^{\rm s}}$
	Organic and total nitrogen	1+^a^, 1–^f^		1+^b^	1+^aa^			
	Available nutrients (phosphorus, potassium)	1+^a^, 1–^f^		1+^b^	2+^aa,ab^		1+^ag^	1+^ab^
	pH	1+^a^, 1–^f^		1+^a^				
	Cation exchange capacity	1 ns^l^						
Biological	Pathogens					1+^ae^		
	Mycorrhizal fungi	3+^m,n,o^, 1–^o^		2+ ^w,x^, 1 ns^z^		2+^w,z^		
	Nitrogen-fixing bacteria	1+^p^, 1–^p^						
	Soil Fauna		1+^u^					1+^ah^
	Fine root biomass	1+^h^, 1 ns^q^	1+^q^		1+^k^			
	Microbial biomass	1 ns^j^				1+^af^		
	Enzymatic activity	1 ns^j^						

*Note:* The references for each reported relationship are provided in this note. ^a^Gavito et al. 2021. ^b^Robison et al. 2015. ^c^Schwartz et al. 2022. ^d^Teixiera et al. 2020. ^e^Bieluczyk et al. 2023. ^f^Robinson et al. 2015. ^g^Gogoi et al. 2020. ^h^Martin et al. 2013. ^i^Ojoatre et al. 2024. ^j^Pantaleão et al. 2024. ^k^Jones et al. 2019. ^l^Poorter et al. 2016. ^m^Schuldt et al. 2023. ^n^Zhang et al. 2021. ^o^Soudzilovskaia et al. 2019. ^p^Batterman et al. 2013. ^q^Hertel et al. 2007. ^r^Lozano-Baez et al. 2021. ${_{.}^{\rm s}}$Marin-Spiotta et al. 2009. ^t^Pandolfo-Paz et al. 2016. ^u^Yang et al. 2014. ^v^Poorter et al. 2021. ^w^Mai et al. 2023. ^x^Peay et al. 2013. ^z^Mueller et al. 2014. ^aa^Campo and Vázquez-Yanes 2004. ^ab^Fisher et al. 2020. ^ac^Zhao et al. 2023. ^ad^Wallwork et al. 2022. ^ae^Adamo et al. 2021. ^af^Bonner et al. 2019. ^ag^Ficetola et al. 2008. ^ah^Cole et al. 2020.

Some established relationships between above- and belowground recovery indicators can serve as a starting point to link patterns of above- and belowground recovery. For example, allometric equations that predict belowground biomass from tree diameter, height, or wood density provide a first approximation for quantifying coarse root stocks (Hertel et al. 2007); however, the appropriate equation may depend on climatic regime and successional stage, as plants invest more in belowground resources under dry conditions and in older forests where water is limiting, and there is higher competition for resources (Waring and Powers 2017). Given these challenges, although allometric equations can provide a rough approximation of belowground biomass, we recommend applying them with caution and prioritizing direct, site-level measurements whenever possible. Both empirical and modeling studies demonstrate tight links between microclimatic conditions and tree or shrub abundance, which typically occur following canopy closure (Lebrija-Trejos et al. 2011, DeFrenne et al. 2021). With increases in aboveground properties, such as tree basal area and leaf area index, during forest recovery, temperatures at the soil surface decline because tree canopies buffer climatic extremes, leading to increased soil moisture during seasonal drought (Schwartz et al. 2022).

Soil physical properties related to bulk density, soil moisture, and hydraulic conductivity (the ratio of overland flow versus infiltration) may change as aboveground biomass, canopy cover, and diversity increase (Teixeira et al. 2020, Lozano-Baez et al. 2021, Poorter et al. 2021), presumably coupled with an increase in coarse root biomass and root enzymes and exudates that increase soil porosity. In addition, a study that explicitly evaluated the extent to which easy-to-measure aboveground indicators correlated with belowground recovery during secondary succession found that soil physical and chemical properties such as bulk density, total soil organic matter, and soil nitrogen concentrations were negatively correlated with both basal area and leaf litter. These results reflect a shift from more degraded sites with compacted soils and lower organic matter and nitrogen to more recovered sites with higher basal area, greater litter accumulation, and improved soil properties (Gavito et al. 2021). Multiple studies also indicate that plant diversity and biomass were positively correlated with fungal diversity in young and old tropical forests, likely because of host specificity and greater resource heterogeneity provided by diverse plant communities (Peay et al. 2013, Schappe et al. 2017, Zhang et al. 2021).

Despite the potential for using commonly collected aboveground data to infer belowground processes and properties, not all belowground properties of interest may be clearly linked with aboveground variables. In fact, across broad areas of tropical forest, there is often no relationship between aboveground biomass or structure and soil properties (Fisher et al. 2020). For example, aboveground biomass is not always correlated with soil carbon (Ojoatre et al. 2024), which is governed by plant productivity and losses of carbon via microbial pathways (Tao et al. 2023). Despite extensive research on tropical forest regeneration, many relationships between above- and belowground recovery have simply not been investigated (table 1). Key gaps include relationships between aboveground properties and belowground indicators such as soil aggregate stability, hydraulic conductivity and infiltration, cation exchange capacity, enzymatic activity, and soil pathogens and fauna. For these cases, gathering more data on belowground properties during tropical forest recovery is necessary. Below, we highlight existing straightforward and scalable approaches to accelerate this process.

## How can we improve our understanding of belowground recovery during restoration?

**Developing a straightforward strategy to measure belowground recovery**—A suite of existing indicators can be used to determine the initial state of soil properties at a restoration site, as well as their recovery, despite the fact that belowground properties are largely overlooked in forest restoration projects (Gatica-Saavedra et al. 2023). To streamline methods for monitoring belowground recovery, we propose a shortlist of six priority indicators to be measured before and after restoration interventions. These indicators capture key physical, chemical, and biological dynamic soil properties in a straightforward and cost-effective way (table 2). Importantly, all six indicators are dynamic (*sensu* Veldkamp et al. 2020), meaning they can change over years to decades following restoration interventions.

**Table 2.** Key approaches for restoration projects to measure recovery of key dynamic soil physical, chemical, and biological properties, what they indicate (before and after intervention), and how to measure them.

Type of property	Soil properties	What it captures	Example method	Important considerations
Physical	Bulk density 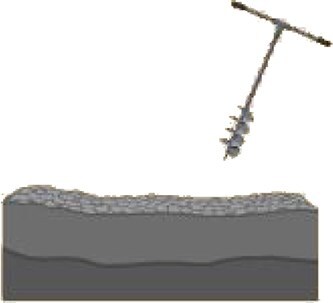	Related directly with soil porosity, soil compaction, erosion resistance, and water and air infiltration rates. Soils with low bulk density have high soil porosity and infiltration rates, and low compaction and erosion resistance. Key parameter for calculating soil carbon stocks.	Collect soil with a corer of known volume at least from 0 to 5 cm depth (or up to 15 cm depth). Trim excess soil from the core ends. Weigh the soil after drying at 105 degrees Celsius (°C) to a stable weight. Divide the dry soil weight by the total soil volume to calculate bulk density.	Ideally, compare measurements taken in a restoration intervention to a reference site to track recovery or take an initial measurement in a restoration area and measure change over time. Refer to methods in (Anderson and Ingram 1994) for more details.
	Aggregate stability 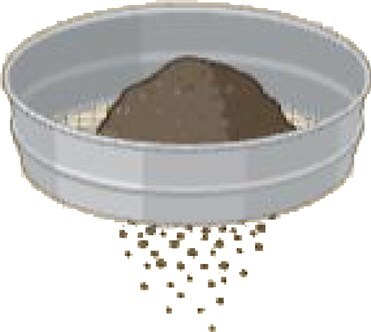	Tracks the recovery of water infiltration rates, decreasing erosion rates, and overall soil health (e.g., nutrient cycling, and biological activity). Soils with high aggregate stability have high infiltration rates and soil health, and experience low erosion.	Remove an intact clod of soil from the 5 cm surface layer. Break apart aggregates and dry overnight. Mount a smartphone 12 cm above an empty plastic dish and place 3 aggregates inside. Fill a second dish with water. Start the SLAKES app, move the second dish filled with water into the camera view, transfer the aggregates to the water filled dish, and take a picture to start the test. After 10 min the app will estimate the aggregate stability index value.	Use clear plastic or glass containers. The mesh should be made from hardware cloth and fit into the top of the container and hold the soil in the top half of the container. Aggregates from soils with poor structure will break apart in water. Refer to the methods in Flynn and colleagues (2020) for more details.
Chemical	Soil organic matter or soil organic carbon 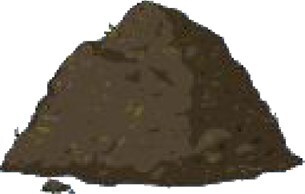	Represents the recovery of soil carbon stocks. It is a fundamental control on soil structure. Soils with high soil organic matter have high water-holding capacity and ecosystem productivity. In practice, it is assumed that soil organic carbon comprises a fixed percentage of soil organic matter (58%) allowing for interconversion of these metrics.	Collect soil with a corer at 0–5 cm depth and air dry. Transfer to plastic Ziploc bags (save approximately 50 grams (g) of soil per sample, some methods require much less) or another suitable container and deliver to a local soil analysis lab.	Recommended analysis methods depend on access to equipment. Loss on ignition or the Walkley–Black method (based on wet oxidation) are used to measure soil organic matter. We suggest focusing on particulate organic matter at early project stages. Elemental analyzers are used to quantify soil organic carbon using dry combustion approaches. Refer to methods in Nelson and Sommers (1996) or to your local analysis lab for more details.
	pH 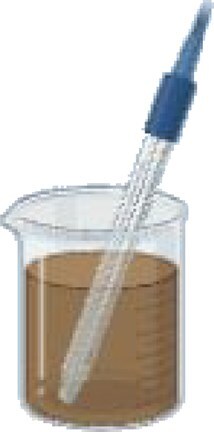	Affects the amount of nutrients and chemicals that are soluble in soil water, and therefore the amount of nutrients available to plants and microbes, and is also correlated with exchangeable base cations. Soils with low pH values (less than 5) have low cation exchange capacity and nutrient availability.	Collect soil with a corer at 0–5 cm depth and air dry it for three days. Take 20 g of the sample and add 50 milliliters of distilled or deionized water. Stir the mixture for 10 minutes. Let it sit for 30 minutes. Start stirring the sample again after 2 minutes. Measure the pH of the supernatant.	Record the first number or wait for a stable reading while using the pH meter Refer to methods in Anderson and Ingram (1994) for more details.
Biological	Decomposition rate 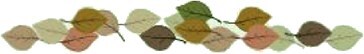	Represents the interactions between soil organisms, physical environmental factors, and resource quality. Soils with high decomposition rates have higher nutrient availability and increase aggregate stability in the long term.	Make decomposition bags with native litter or use tea bags (1.6–1.8 g of total biomass and a mesh size of approximately 0.25 millimeters (mm). Weigh the bags and bury them (8-cm-deep holes, approximately 15 cm apart. Keep the labels visible. Collect the decomposition bags after approximately 60 days. Remove adhering soil particles and dry the bags at 70°C for 48 hours in the sun for 3 or 4 days. Take the litter or tea out of the bag and weigh it. Calculate the decomposition rate following Keuskamp and colleagues (2013).	Native litter should be used when possible. Record the start and end date of the experiment. Avoid using water to remove the soil particles, this can cause loss of material from the bag Refer to (Keuskamp et al., 2013) for more details.
	Macrofaunal abundance 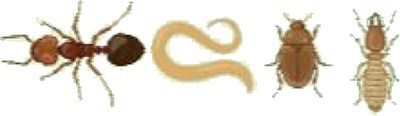	Captures the diversity of macrofauna, their potential role in processes such as decomposition and nutrient cycling activity. Soils with a high diversity of macrofauna tend to have high soil structure and nutrient availability	Remove litter from a 25 cm quadrant and retain for sorting. Use a spade to cut down a few cm outside the quadrant, then dig a 20 × 30 cm deep trench around it. This targets soil macrofauna (more than 2 mm), such as earthworms and arthropods (e.g., Coleoptera, Isopoda, Hymenoptera), important for decomposition and nutrient cycling Collect all invertebrates longer than 10 mm excavated from the trench. Divide the quadrant into three layers (0–10, 10–20, and 20-30 cm), to capture variation in vertical distribution. Sort the soil and litter in trays. Preserve the invertebrates in 4% formaldehyde. Keep earthworms separate from other groups. Record abundance and fresh (preserved) weight of invertebrates in the litter and each of the three strata.	For a 1-hectare area, randomly assign five points for quadrat locations. Refer to methods in Anderson and Ingram (1994) for more details.

*Note:* See the citations in the table for more detailed methods for each soil property.

For each indicator, we recommend measuring initial preintervention values, followed by postintervention recovery at regular intervals, such as every 5 years at a minimum or more frequently if feasible (Lal 1994, Smith 2004, Silva-Olaya et al. 2025). Some indicators may vary seasonally following the peak of biological activity (e.g., pH, decomposition rates, macrofauna abundance; Lal 1994) and should be sampled during the growing season when biological activity is highest (Amazonas et al. 2017, Pajares et al. 2018). Where possible, additional sampling in the nongrowing season is recommended to better understand how seasonal changes influence above- and belowground recovery (Silva et al. 2024). To account for high spatial heterogeneity in tropical soil properties, we recommend measuring each of the properties below in at least three locations (ideally 5–10) per sampling plot, spaced at least 5 meters apart and distributed along a transect, or, ideally, randomly distributed or randomly stratified within the sampling plot (following van der Sande et al. 2023). Importantly, sampling intensity should reflect local variation rather than total project size—that is, a small, very heterogeneous 10-hectare site may require similar sampling effort as a 10,000-hectare project. Optimal sampling areas (i.e., how many plots to install) for measuring belowground properties in a restoration context still need to be developed, but as a starting rule of thumb, Londe and colleagues (2022) generally found that 2%–4% of a project's area should be sampled to robustly track recovery of aboveground indicators in tropical restoration. We recommend belowground indicators be collected from subplots or transects nested within these sampling plots to ensure feasibility and allow for integration with aboveground monitoring. Finally, we recommend measuring fewer properties with higher intensity (i.e., more samples, higher frequency) rather than more properties with less sampling effort (i.e., fewer samples, lower frequency).

For physical properties, we recommend first measuring bulk density, because of its ease of measurement, followed by aggregate stability. For the first four properties in the shortlist, we recommend taking measurements in the top 10 centimeters (cm) of soil—or slightly deeper (10–20 cm) in areas where intensive agriculture has significantly disturbed the surface layer (table 2; Keller and Or 2022). This top layer is highly sensitive to disturbance, changes rapidly during the first 5 years of forest recovery and holds a relatively high concentration of soil carbon (Cusack et al. 2018, Poorter et al. 2021, Witzgall et al. 2021, van der Sande et al. 2023). Both soil bulk density and aggregate stability are negatively affected by soil compaction and covary with soil infiltration and water holding capacity. High aggregate stability increases infiltration and water holding capacity, whereas a high bulk density decreases both variables (Horn et al. 1995). Bulk density (i.e., dry mass per unit volume) is a low-tech indicator of soil physical health (Hatten and Liles 2019) that is necessary to accurately estimate soil carbon stocks. Increases in aggregate stability have been linked to the recovery of factors such as fine root biomass and soil organic carbon during forest succession (Xiao et al. 2020), and aggregate stability can now be measured quickly and inexpensively using a novel smartphone application that analyzes images of soil peds before and after wetting (SLAKES; Flynn et al. 2020). If resources allow, measuring the recovery of soil organic horizons (i.e., depth of the organic horizon, approximately 0–20 cm), particularly the forest floor (i.e., Oi horizon, USDA-SSDS; O horizon FAO-IUSS), can be a good proxy indicator for soil moisture and nutrient availability (Cusack and Montagnini 2004).

For soil chemical properties, we suggest first measuring soil organic matter, focusing on the particulate organic matter fraction, which is more sensitive to disturbance and exhibits a high turnover (Lehmann et al. 2001). pH is another inexpensive indicator to assess soil nutrient availability, the impact of previous land use (e.g., burning, fertilizers), and cation exchange capacity (Neina 2019). If resources allow, quantifying total soil carbon and nitrogen can track recovery of soil biogeochemical cycles, and quantifying soil phosphorus can assess initial nutrient availability and facilitate prescribing restoration interventions, although the availability of these nutrients is usually closely correlated with total soil organic matter and pH (Hatten and Liles 2019, Sullivan et al. 2019), so we recommend prioritizing those indicators first.

For soil biological recovery we first recommend tracking decomposition rates using decomposition bags to evaluate the activity of saprophytic microbes (i.e., some bacteria and white rot, brown rot, and soft rot fungi) through their role in organic matter decomposition (McGuire and Treseder 2010, Keuskamp et al. 2013, Sarneel et al. 2024). This can be measured by placing groups of nylon mesh bags, ideally filled with a consistent mix of leaf litter from local native trees, or a common substrate if necessary (e.g., tea bags), into the field. The bags should then be collected over time, or at a specific point in strongly seasonal systems, to measure mass loss. For a second biological property, macrofauna abundance can be evaluated via earthworm or arthropod (e.g., ants and termites) counts in soils (DeLuca et al. 2019) and could potentially be facilitated by emerging ecoacoustics methods (Maeder et al. 2022) or more costly techniques such as eDNA (Liddicoat et al. 2022). Both decomposition rates and macrofauna abundance are expected to increase in the first few years after a site has been restored (Serra et al. 2021). More costly and technologically involved measurements of soil properties, that are also very useful if the capacity exists, include quantifying soil microbial diversity and biomass (i.e., through phospholipid and neutral fatty acids analysis), root mycorrhizal colonization, fine root biomass soil enzymatic activity, and soil respiration.

To ensure consistency and comparability in reporting, especially when assessing restoration outcomes across different sites, we recommend clearly reporting the methods used for data collection and using the following units for the six indicators in table 2: *Physical properties*, for which bulk density should be reported per unit volume (e.g., in grams per cubic centimeter), and aggregate stability is typically expressed as a percentage or index. *Chemical properties*, where organic matter and soil carbon should be reported as a percentage of soil mass (e.g., as a percentage or in milligrams per kilogram); however, when estimating total soil carbon stocks, these values should be converted to a per-volume basis using bulk density and sampling depth. pH should be reported using standard, unitless measurements. And *biological properties*, for which both decomposition rate and macrofauna abundance should be reported per unit area or volume, depending on the sampling method.

### Emerging technologies may assist in monitoring belowground recovery

We suggest prioritizing measurement of the six top-priority metrics to thoroughly track belowground recovery, but recent advances in remote and near sensing monitoring may also be helpful if project resources allow. Remote sensing techniques such as passive microwave or active radar can measure changes in surface soil moisture at initial restoration stages when the canopy is open (Entekhabi et al. 2010), but field measurements are currently needed after this stage to track recovery. Similarly, hyperspectral measurements can predict mycorrhizal associations of canopy tree species across large spatial scales. Changes in these associations over time can be linked to shifts in species composition and nutrient cycling, making them a useful tool for monitoring the large-scale impacts of forest restoration. However, these relationships require further refinement in tropical ecosystems (Sousa et al. 2021). For *in*  *situ* near-sensing ecoacoustics, the study of all sounds emitted in a location (Sueur and Farina 2015), has emerged as an efficient tool to provide a rapid assessment of belowground recovery during tropical forest restoration. For example, taxa richness of soil faunal communities can be predicted using ecoacoustics metrics (Brandhorst-Hubbard et al. 2001, Maeder et al. 2022, Robinson et al. 2023), and ecoacoustics can also predict soil aggregate stability (Quintanilla‐Tornel 2017).

## A path forward for holistic design and assessments of tropical forest restoration

**Looking toward practice to understand and evaluate the impacts of interventions on belowground recovery**—Understanding the initial belowground conditions at a given site can set the stage for forest recovery by guiding how and why soil amendments should be incorporated into assisted restoration interventions. Incorporating soil management practices and plant belowground traits, including potential associations with microbial symbionts, into restoration interventions can help restore feedback loops between above- and belowground processes. However, best practices for the use of soil amendments and design of species mixes that consider belowground traits are poorly defined.

Integrating various soil amendments into restoration design has the potential to enhance both above- and belowground outcomes. In areas with high water deficits, adding organic materials (e.g., straw, mulch found in the area) or hydrogels directly to planted seedlings holes may enhance water retention and improve root access to water in both temperate (Chirino et al. 2011) and tropical regions (e.g., Werden et al. 2018). For low-fertility tropical soils, chemical amendments directly added to the soil around seedlings, such as inorganic fertilizers or compost, can increase nutrient availability (Cuenca et al. 1997), whereas applying liming and biochar can raise the pH of acidic soils, facilitating plant establishment in systems where pH has been modified (Thomas and Gale 2015). In degraded soils that have experienced prolonged land use or are far from mature forests, biological amendments, such as native litter addition, can accelerate the recovery of soil biota and boost nutrient availability in nuclei and islands (Martins 2018, Singh Rawat et al. 2023), without causing a substantial impact on donor forests as long as litter removal is limited to the short term (Sayer 2006). Similarly, inoculating seedlings with local mycorrhizal fungi or symbiotic nitrogen-fixing bacteria can promote seedling establishment (e.g., Maltz and Treseder 2015, Lance et al. 2019) and growth (Neuenkamp et al. 2019). Emerging approaches, such as the development of designer microbiomes, hold promise for addressing specific deficiencies in soil microbial communities, although these methods remain cost intensive and need further evaluation (Robinson et al. 2024). Despite the potential of soil amendments to improve assisted restoration outcomes (Werden et al. 2024), replicated experiments are needed to address critical questions, including how to apply them (e.g., broadly or targeted), the optimal quantities, and whether reapplication is necessary—and, if so, how often.

In addition to using soil amendments, assisted restoration outcomes can be improved through careful species selection at the outset of projects. For instance, seedling survival can be optimized by planting species with specific belowground traits. To this end, early successional species that tend to be deeply-rooted typically have higher survival rates in forests that experience seasonal drought (Baraloto et al. 2010, Paz et al. 2015, Cheesman et al. 2018, Werden et al. 2022). Further targeted restoration experimentation is necessary to understand how belowground plant traits can be leveraged to improve initial restoration outcomes (e.g., plant establishment and growth), and how planting species with specific suites of traits are linked explicitly to the recovery of soil physical (e.g., with deposition of organic matter), chemical (e.g., through symbiotic relationships with nitrogen-fixing bacteria or mycorrhizal fungi on plant roots), and biological (e.g., through mycorrhizal symbiosis) properties.

### A need to unlock the ability to monitor belowground recovery over time

Although there are straightforward methods available to measure how above- and belowground properties shift along restoration trajectories, time series tracking belowground recovery remain scarce. There is a need to further quantify both the initial state of belowground properties before intervention and then determine how long they take to recover (Veldkamp et al. 2020). Recovery time depends on the type of ecosystem, previous land use, soil type, and disturbance intensity. For instance, soil nitrogen may recover within a decade, whereas properties such as pH, soil organic matter, and decomposition rates can take several decades to approach values typical of soils in mature forest (Veldkamp et al. 2020, Poorter et al. 2021, Van der Sande et al. 2023). This can be especially important in early restoration stages (years 5–10) for aspects such as soil microbial recovery (Silva et al. 2024), which can either positively or negatively affect assisted restoration success—for instance, by affecting seedling survival (McCulloch et al. 2024). Therefore, integrating belowground metrics into monitoring programs that already assess aboveground recovery is essential, especially when the two trajectories are decoupled, as has been observed for properties such as carbon stocks (Jones et al. 2019) and ecohydrological processes (Lloyd et al. 2013).

Some efforts have been made to determine how restoration projects are measuring belowground recovery and where gaps remain (e.g., Gatica-Saavedra et al. 2023). However, we emphasize that the indicators and protocols used must be streamlined and standardized for the measurement of belowground recovery to be realistic in most restoration projects. To monitor belowground recovery efficiently and thoroughly across restoration projects, the barriers to entry must be kept low by minimizing costs, ensuring that properties are easy to measure, and providing standardized monitoring protocols for measurement and analysis (e.g., those in table 2). In this regard, the restoration community could learn from the global land outlook and emerging biodiversity monitoring networks (UNCCD 2022, Gonzalez et al. 2023) and should ensure that belowground recovery is prioritized in the Framework for Ecosystem Restoration Monitoring of the UN Decade on Ecosystem Restoration (UN Decade on Ecosystem Restoration 2024)*.*

### Leveraging ecosystem modeling to link above- and belowground recovery, optimize restoration strategies, and predict outcomes at scale

Although considerable efforts have been devoted to developing restoration prioritization (e.g., Strassburg et al. 2020, Löfqvist et al. 2023) and opportunity maps (e.g., Brancalion et al. 2019), these efforts have largely focused on aboveground outcomes. Ecosystem models remain an underused tool that could provide critical insights into how different restoration interventions influence tropical forest recovery trajectories, including the links—or a lack thereof—between above- and belowground recovery (e.g., those explored in table 1). Moreover, a major challenge in restoration is ensuring long-term project sustainability, particularly under a changing climate. Simply restoring native species may not guarantee productivity and resiliency when the future is unlike the past (Simonson et al. 2021). Ecosystem models can help project restoration outcomes, offering valuable perspectives on both the potential for above- and belowground recovery and the future behavior of current restoration decisions under varying climate scenarios (Fisher et al. 2014, Terrer et al. 2021, Koch and Kaplan 2022).

For example, ecosystem models have successfully identified strategies to mitigate soil phosphorus loss during land-use change, supporting the subsequent recovery of soil processes (Nagy et al. 2017). In addition, terrestrial biosphere models (e.g., Longo et al. 2019) could be used to track plant demographic dynamics within restoration interventions, incorporating age cohorts of plant functional types (e.g., nitrogen fixers, successional stage strategists). Finally, models that integrate both ecological and economic dimensions can help assess the overall viability of a restoration project and identify when interventions are cost effective (Bodini et al. 2024). By leveraging such tools, we can create a more complete picture of above- and belowground recovery dynamics and scale site-level information to broader landscapes undergoing specific restoration intervention (e.g., Medvigy et al. 2019).

## Conclusions

The state of belowground properties can dictate the pace and trajectory of ecosystem recovery, and the goal of restoration interventions is to catalyze this process. Monitoring both above- and belowground ecosystem properties is necessary to gain a comprehensive picture of forest ecosystem recovery and their capacity to provide ecosystem services. As restoration initiatives are scaled up, it is critical to expand our knowledge of above- and belowground recovery dynamics (table 1) to robustly assess whether interventions are meeting the initial restoration goals. Achieving this will require that scientists and practitioners conduct field trials to explore understudied connections between above- and belowground recovery, build novel ecosystem models that integrate restoration interventions, and adopt standardized, low-cost methods to monitor belowground recovery. To gather the data needed to do so, we recommend two top-priority indicators for each dynamic soil property group—physical, chemical, and biological—to track soil recovery in tropical forest restoration in a straightforward manner (table 2), along with additional indicators to consider when resources allow. These recommendations serve as a blueprint to harmonize the integration of both above- and belowground recovery into restoration design, and guide initiatives such as the United Nations Standards of Practice to Guide Ecosystem Restoration (FAO et al. 2023).

## Supplementary Material

biaf097_Supplementary_File
