# Supplementary material for: Integrating belowground recovery into tropical forest restoration design and monitoring
Source: Bioscience. 2025 Jul 14;75(11):937–52. doi: 10.1093/biosci/biaf097 (PMC12650528; doi:10.1093/biosci/biaf097)
Supplement: biaf097_Supplementary_File [file biaf097_supplementary_file.docx]

| **Toro, Werden et al. Supplement** | | | | | | | |
| --- | --- | --- | --- | --- | --- | --- | --- |
| *Search in Web of Science Core Collection:* above* AND below* AND (recover* OR restor* OR rehabilitat*) AND tropic* AND forest* (Topic), on July 31, 2024 and relevant references found in the citation lists of the "In scope" papers. | | | | | | | |
| **In scope/included in Table 1:** Direct comparison of above- and belowground recovery in tropical wet and/or dry forest | | | | | |  |  |
| **ID** | **Authors** | **Article Title** | | **Source Title** | **ISSN** | **Publication Year** | **DOI** |
| 1 | Adamo, I; Ortiz-Malavasi, E; Chazdon, R; Chaverri, P; ter Steege, H; Geml, J | Soil Fungal Community Composition Correlates with Site-Specific Abiotic Factors, Tree Community Structure, and Forest Age in Regenerating Tropical Rainforests | | Biology |  | 2021 | 10.3390/biology10111120 |
| 2 | Batterman, SA; Hedin, LO; van Breugel, M; Ransijn, J; Craven, DJ; Hall, JS | Key role of symbiotic dinitrogen fixation in tropical forest secondary succession | | Nature | 0028-0836 | 2013 | 10.1038/nature12525 |
| 3 | Bieluczyk, W; Asselta, FO; Navroski, D; Gontijo, JB; Venturini, AM; Mendes, LW; Simon, CP; de Camargo, PB; Tadini, AM; -Neto, LM; Bendassolli, JA; Rodrigues, RR; van der Putten, WH; Tsai, SM | Linking above and belowground carbon sequestration, soil organic matter properties, and soil health in Brazilian Atlantic Forest restoration | | Journal Of Environmental Management | 0301-4797 | 2023 | 10.1016/j.jenvman.2023.118573 |
| 4 | Bonner, MTL; Herbohn, J; Gregorio, N; Pasa, A; Avela, MS; Solano, C; Moreno, MOM; Almendras-Ferraren, A; Wills, J; Shoo, LP; Schmidt, S | Soil organic carbon recovery in tropical tree plantations may depend on restoration of soil microbial composition and function | | Geoderma | 0016-7061 | 2019 | 10.1016/j.geoderma.2019.06.017 |
| 5 | Campo, J; Vázquez-Yanes, C | Effects of nutrient limitation on aboveground carbon dynamics during tropical dry forest regeneration in Yucatan, Mexico | | Ecosystems | 1432-9840 | 2004 | 10.1007/s10021-003-0249-2 |
| 6 | Gavito, ME; Paz, H; Barragán, F; Siddique, I; Arreola-Villa, F; Pineda-García, F; Balvanera, P | Indicators of integrative recovery of vegetation, soil and microclimate in successional fields of a tropical dry forest | | Forest Ecology and Management | 0378-1127 | 2021 | 10.1016/j.foreco.2020.118526 |
| 7 | Gogoi, A; Sahoo, UK; Saikia, H | Vegetation and ecosystem carbon recovery following shifting cultivation in Mizoram-Manipur-Kachin rainforest eco-region, Southern Asia | | Ecological Processes |  | 2020 | 10.1186/s13717-020-00225-w |
| 8 | Hertel, D; Leuschner, C; Harteveld, M; Wiens, M | Fine root mass, distribution and regeneration in disturbed primary forests and secondary forests of the moist tropics | | Stability of Tropical Rainforest Margins: Linking Ecological, Economic and Social Constraints of Land Use and Conservation | 1863-5520 | 2007 |  |
| 9 | Jones, IL; DeWalt, SJ; Lopez, OR; Bunnefeld, L; Pattison, Z; Dent, DH | Above- and belowground carbon stocks are decoupled in secondary tropical forests and are positively related to forest age and soil nutrients respectively | | Science of The Total Environment | 0048-9697 | 2019 | 10.1016/j.scitotenv.2019.133987 |
| 10 | Lozano-Baez, SE; Domínguez-Haydar, Y; Zwartendijk, BW; Cooper, M; Tobón, C; Di Prima, S | Contrasts in Top Soil Infiltration Processes for Degraded vs. Restored Lands. A Case Study at the Perija Range in Colombia | | Forests |  | 2021 | 10.3390/f12121716 |
| 11 | Mai, SW; Mao, H; Jiang, YM; Huang, T; Yang, Q; Xing, GT; Wang, XF; Yang, H; Liu, WJ | Characteristics of the soil arbuscular mycorrhizal fungal community along succession stages in tropical forest and its driving factors | | Frontiers In Environmental Science |  | 2023 | 10.3389/fenvs.2022.1110655 |
| 12 | Marin-Spiotta, E; Silver, WL; Swanston, CW; Ostertag, R | Soil organic matter dynamics during 80 years of reforestation of tropical pastures | | Global Change Biology | 1354-1013 | 2009 | 10.1111/j.1365-2486.2008.01805.x |
| 13 | Martin, PA; Newton, AC; Bullock, JM | Carbon pools recover more quickly than plant biodiversity in tropical secondary forests | | Proceedings of The Royal Society B-Biological Sciences | 0962-8452 | 2013 | 10.1098/rspb.2013.2236 |
| 14 | Mueller, RC; Paula, FS; Mirza, BS; Rodrigues, JLM; Nüsslein, K; Bohannan, BJM | Links between plant and fungal communities across a deforestation chronosequence in the Amazon rainforest | | Isme Journal | 1751-7362 | 2014 | 10.1038/ismej.2013.253 |
| 15 | Ojoatre, S; Barlow, J; Jacobs, SR; Rufino, MC | Recovery of aboveground biomass, soil carbon stocks and species diversity in tropical montane secondary forests of East Africa | | Forest Ecology and Management | 0378-1127 | 2024 | 10.1016/j.foreco.2023.121569 |
| 16 | Pandolofo-Paz, C; Goosem, M; Bird, M; Preece, N; Goosem, S; Fensham, R; Laurance, S | Soil types influence predictions of soil carbon stock recovery in tropical secondary forests | | Forest Ecology and Management | 0378-1127 | 2016 | 10.1016/j.foreco.2016.06.007 |
| 17 | Pantaleao, LC; de Moraes, LFD; Cesário, FV; Moser, P; Dias, ATC; Amorim, TA; Sansevero, JBB | Linking plant functional traits to soil properties in tropical forest restoration | | Forest Ecology and Management | 0378-1127 | 2024 | 10.1016/j.foreco.2024.121976 |
| 18 | Paoli, GD; Curran, LM; Slik, JWF | Soil nutrients affect spatial patterns of aboveground biomass and emergent tree density in southwestern Borneo | | Oecologia | 0029-8549 | 2008 | 10.1007/s00442-007-0906-9 |
| 19 | Peay, KG ; Baraloto, C; Fine, PVA | Strong coupling of plant and fungal community structure across western Amazonian rainforests | | The Isme Journal | 1852–1861 | 2013 | 10.1038/ismej.2013.66 |
| 20 | Poorter, L; Bongers, F; Aide, TM; Almeyda Zambrano, AM; Balvanera, P; Becknell, JM; Boukili, V; Brancalion, PHS; Broadbent, EN; Chazdon, RL; Craven, D; de Almeida-Cortez, JS; Cabral, GAL; de Jong, BHJ; Denslow, JS; Dent, DH; DeWalt, SJ; Dupuy, JM; Durán, SM; Espírito-Santo, MM; Fandino, MC; César, RG; Hall, JS; Hernandez-Stefanoni, JL; Jakovac, CC; Junqueira, AB; Kennard, D; Letcher, SG; Licona, JC; Lohbeck, M; Marin-Spiotta, E; Martínez-Ramos, M; Massoca, P; Meave, JA; Mesquita, R; Mora, F; Muñoz, R; Muscarella, R; Nunes, YRF; Ochoa-Gaona, S; de Oliveira, AA; Orihuela-Belmonte, E; Peña-Claros, M; Pérez-García, EA; Piotto, D; Powers, JS; Rodríguez-Velázquez, J; Romero-Pérez, IE; Ruíz, J; Saldarriaga, JG; Sanchez-Azofeifa, A; Schwartz, NB; Steininger, MK; Swenson, NG; Toledo, M; Uriarte, M; van Breugel, M; van der Wal, H; Veloso, MDM; Vester, HFM; Vicentini, A; Vieira, ICG; Bentos, TV; Williamson, GB; Rozendaal, DMA | Biomass resilience of Neotropical secondary forests | | Nature | 0028-0836 | 2016 | 10.1038/nature16512 |
| 21 | Poorter, L; Craven, D; Jakovac, CC; van der Sande, MT; Amissah, L; Bongers, F; Chazdon, RL; Farrior, CE; Kambach, S; Meave, JA; Muñoz, R; Norden, N; Rüger, N; van Breugel, M; Zambrano, AMA; Amani, B; Andrade, JL; Brancalion, PHS; Broadbent, EN; de Foresta, H; Dent, DH; Derroire, G; DeWalt, SJ; Dupuy, JM; Durán, SM; Fantini, AC; Finegan, B; Hernández-Jaramillo, A; Hernández-Stefanoni, JL; Hietz, P; Junqueira, AB; N'dja, JK; Letcher, SG; Lohbeck, M; López-Camacho, R; Martínez-Ramos, M; Melo, FPL; Mora, F; Müller, SC; N'Guessan, AE; Oberleitner, F; Ortiz-Malavassi, E; Pérez-García, EA; Pinho, BX; Piotto, D; Powers, JS; Rodríguez-Buriticá, S; Rozendaal, DMA; Ruíz, J; Tabarelli, M; Teixeira, HM; Sampaio, EVDB; van der Wal, H; Villa, PM; Fernandes, GW; Santos, BA; Aguilar-Cano, J; de Almeida-Cortez, JS; Alvarez-Davila, E; Arreola-Villa, F; Balvanera, P; Becknell, JM; Cabral, GAL; Castellanos-Castro, C; de Jong, BHJ; Nieto, JE; Espírito-Santo, MM; Fandino, MC; García, H; García-Villalobos, D; Hall, JS; Idárraga, A; Jiménez-Montoya, J; Kennard, D; Marín-Spiotta, E; Mesquita, R; Nunes, YRF; Ochoa-Gaona, S; Peña-Claros, M; Pérez-Cárdenas, N; Rodríguez-Velázquez, J; Villanueva, LS; Schwartz, NB; Steininger, MK; Veloso, MDM; Vester, HFM; Vieira, ICG; Williamson, GB; Zanini, K; Hérault, B | Multidimensional tropical forest recovery | | Science | 0036-8075 | 2021 | 10.1126/science.abh3629 |
| 22 | Robinson, SJB; van den Berg, E; Meirelles, GS; Ostle, N | Factors influencing early secondary succession and ecosystem carbon stocks in Brazilian Atlantic Forest | | Biodiversity and Conservation | 0960-3115 | 2015 | 10.1007/s10531-015-0982-9 |
| 23 | Schuldt, A; Liu, XJ; Buscot, F; Bruelheide, H; Erfmeier, A; He, JS; Klein, AM; Ma, KP; Scherer-Lorenzen, M; Schmid, B; Scholten, T; Tang, ZY; Trogisch, S; Wirth, C; Wubet, T; Staab, M | Carbon-biodiversity relationships in a highly diverse subtropical forest | | Global Change Biology | 1354-1013 | 2023 | 10.1111/gcb.16697 |
| 24 | Schwartz, NB; Medvigy, D; Tijerin, J; Pérez-Aviles, D; Rivera-Polanco, D; Pereira, D; Vargas, GG; Werden, L; Du, D; Arnold, L; Powers, JS | Intra-annual variation in microclimatic conditions in relation to vegetation type and structure in two tropical dry forests undergoing secondary succession | | Forest Ecology and Management | 0378-1127 | 2022 | 10.1016/j.foreco.2022.120132 |
| 25 | Teixeira, HM; Cardoso, IM; Bianchi, FJJA; Silva, AD; Jamme, D; Peña-Claros, M | Linking vegetation and soil functions during secondary forest succession in the Atlantic Forest | | Forest Ecology and Management | 0378-1127 | 2020 | 10.1016/j.foreco.2019.117696 |
| 26 | Wallwork, A; Banin, LF; Dent, DH; Skiba, U; Sayer, E | Soil carbon storage is related to tree functional composition in naturally regenerating tropical forests | | Functional Ecology | 0269-8463 | 2022 | 10.1111/1365-2435.14221 |
| 27 | Zhang, J; Quan, CX; Ma, LL; Chu, GW; Liu, ZF; Tang, XL | Plant community and soil properties drive arbuscular mycorrhizal fungal diversity: A case study in tropical forests | | Soil Ecology Letters | 2662-2289 | 2021 | 10.1007/s42832-020-0049-z |
| 28 | Zhao, ST; Zhao, XY; Li, YL | Relationship between the trait response of aboveground and belowground parts of dominant plant species to groundwater depth change in Horqin Sandy Land, eastern China | | Ecological Indicators | 1470-160X | 2023 | 10.1016/j.ecolind.2023.111001 |
| **Out Of Scope/Excluded From Table 1:** No Direct Comparison Of Above- And Belowground Recovery, OR Not In Tropical Wet And/Or Dry Forest (E.G., Mangroves Excluded) | | | | | | | |
| **ID** | **Authors** | **Article Title** | **Source Title** | | **ISSN** | **Publication Year** | **DOI** |
| 29 | Abbas, S; Nichol, JE; Fischer, GA | Mapping and assessment of impacts of cold and frost on secondary forest in the marginally tropical landscape of Hong Kong | Agricultural and Forest Meteorology | | 0168-1923 | 2017 | 10.1016/j.agrformet.2016.10.008 |
| 30 | Adame, MF; Kauffman, JB; Medina, I; Gamboa, JN; Torres, O; Caamal, JP; Reza, M; Herrera-Silveira, JA | Carbon Stocks of Tropical Coastal Wetlands within the Karstic Landscape of the Mexican Caribbean | Plos One | | 1932-6203 | 2013 | 10.1371/journal.pone.0056569 |
| 31 | Adinugroho, WC; Krisnawati, H; Imanuddin, R; Siregar, CA; Weston, CJ; Volkova, L | Developing biomass allometric equations for small trees in mixed-species forests of tropical rainforest ecozone | Trees Forests and People | |  | 2023 | 10.1016/j.tfp.2023.100425 |
| 32 | Adinugroho, WC; Prasetyo, LB; Kusmana, C; Krisnawati, H; Weston, CJ; Volkova, L | Recovery of Carbon and Vegetation Diversity 23 Years after Fire in a Tropical Dryland Forest of Indonesia | Sustainability | |  | 2022 | 10.3390/su14126964 |
| 33 | Aguiar, BAD; Lopes, CGR; de Sousa, GM; Medeiros, MJLE; da Silva, KA; Araújo, ED | Effect of simulated extreme rainfall on the vegetative phenology of perennial and annual herbaceous plants from a Brazilian dry forest | Plant Biology | | 1435-8603 | 2024 | 10.1111/plb.13674 |
| 34 | Alongi, DM | Carbon sequestration in mangrove forests | Carbon Management | | 1758-3004 | 2012 | 10.4155/CMT.12.20 |
| 35 | Ammondt, SA; Litton, CM | Competition between Native Hawaiian Plants and the Invasive Grass Megathyrsus maximus: Implications of Functional Diversity for Ecological Restoration | Restoration Ecology | | 1061-2971 | 2012 | 10.1111/j.1526-100X.2011.00806.x |
| 36 | Anthelme, F; Lincango, J; Gully, C; Duarte, N; Montúfar, R | How anthropogenic disturbances affect the resilience of a keystone palm tree in the threatened Andean cloud forest? | Biological Conservation | | 0006-3207 | 2011 | 10.1016/j.biocon.2010.12.025 |
| 37 | Argibay, DS; Sparacino, J; Espindola, GM | A long-term assessment of fire regimes in a Brazilian ecotone between seasonally dry tropical forests and savannah | Ecological Indicators | | 1470-160X | 2020 | 10.1016/j.ecolind.2020.106151 |
| 38 | ASBJORNSEN, H; MONTAGNINI, F | VESICULAR-ARBUSCULAR MYCORRHIZAL INOCULUM POTENTIAL AFFECTS THE GROWTH OF STRYPHNODENDRON-MICROSTACHYUM SEEDLINGS IN A COSTA-RICAN HUMAN TROPICAL LOWLAND | Mycorrhiza | | 0940-6360 | 1994 | 10.1007/s005720050040 |
| 39 | Asbjornsen, H; Velázquez-Rosas, N; García-Soriano, R; Gallardo-Hernández, C | Deep ground fires cause massive above- and below-ground biomass losses in tropical montane cloud forests in Oaxaca, Mexico | Journal of Tropical Ecology | | 0266-4674 | 2005 | 10.1017/S0266467405002373 |
| 40 | Asmelash, F; Bekele, T; Birhane, E | The Potential Role of Arbuscular Mycorrhizal Fungi in the Restoration of Degraded Lands | Frontiers in Microbiology | |  | 2016 | 10.3389/fmicb.2016.01095 |
| 41 | BELLINGHAM, PJ; TANNER, EVJ; HEALEY, JR | SPROUTING OF TREES IN JAMAICAN MONTANE FORESTS, AFTER A HURRICANE | Journal of Ecology | | 0022-0477 | 1994 | 10.2307/2261440 |
| 42 | Bellingham, PJ; Tanner, EVJ; Rich, PM; Goodland, TCR | Changes in light below the canopy of a Jamaican montane rainforest after a hurricane | Journal of Tropical Ecology | | 0266-4674 | 1996 | 10.1017/S0266467400009883 |
| 43 | Berenguer, E; Ferreira, J; Gardner, TA; Aragao, LEOC; De Camargo, PB; Cerri, CE; Durigan, M; De Oliveira, RC; Vieira, ICG; Barlow, J | A large-scale field assessment of carbon stocks in human-modified tropical forests | Global Change Biology | | 1354-1013 | 2014 | 10.1111/gcb.12627 |
| 44 | Bhomia, RK; MacKenzie, RA; Murdiyarso, D; Sasmito, SD; Purbopuspito, J | Impacts of land use on Indian mangrove forest carbon stocks: Implications for conservation and management | Ecological Applications | | 1051-0761 | 2016 | 10.1890/15-2143 |
| 45 | Birhane, E; Aregawi, K; Giday, K | Changes in arbuscular mycorrhiza fungi spore density and root colonization of woody plants in response to exclosure age and slope position in the highlands of Tigray, Northern Ethiopia | Journal of Arid Environments | | 0140-1963 | 2017 | 10.1016/j.jaridenv.2017.03.002 |
| 46 | Birke, A; Aluja, M | Anastrepha ludens and Anastrepha serpentina (Diptera: Tephritidae) Do Not Infest Psidium guajava (Myrtaceae), but Anastrepha obliqua Occasionally Shares This Resource With Anastrepha striata in Nature | Journal of Economic Entomology | | 0022-0493 | 2011 | 10.1603/EC11042 |
| 47 | Bomfim, B; Pinagé, ER; Emmert, F; Kueppers, LM | Improving sustainable tropical forest management with voluntary carbon markets | Plant and Soil | | 0032-079X | 2022 | 10.1007/s11104-021-05249-5 |
| 48 | Brahma, B; Nath, AJ; Das, AK | Managing rubber plantations for advancing climate change mitigation strategy | Current Science | | 0011-3891 | 2016 | 10.18520/cs/v110/i10/2015-2019 |
| 49 | Brearley, FQ | Below-ground secondary succession in tropical forests of Borneo | Journal of Tropical Ecology | | 0266-4674 | 2011 | 10.1017/S0266467411000149 |
| 50 | Brown, S; Schroeder, P; Birdsey, R | Aboveground biomass distribution of US eastern hardwood forests and the use of large trees as an indicator of forest development | Forest Ecology and Management | | 0378-1127 | 1997 | 10.1016/S0378-1127(97)00044-3 |
| 51 | Bustamante, MMC; Silva, JS; Scariot, A; Sampaio, AB; Mascia, DL; Garcia, E; Sano, E; Fernandes, GW; Durigan, G; Roitman, I; Figueiredo, I; Rodrigues, RR; Pillar, VD; de Oliveira, AO; Malhado, AC; Alencar, A; Vendramini, A; Padovezi, A; Carrascosa, H; Freitas, J; Siqueira, JA; Shimbo, J; Generoso, LG; Tabarelli, M; Biderman, R; Salomao, RD; Valle, R; Junior, B; Nobre, C | Ecological restoration as a strategy for mitigating and adapting to climate change: lessons and challenges from Brazil | Mitigation And Adaptation Strategies for Global Change | | 1381-2386 | 2019 | 10.1007/s11027-018-9837-5 |
| 52 | Cai, HY; Lu, HF; Tian, Y; Liu, ZF; Huang, Y; Jian, SG | Effects of invasive plants on the health of forest ecosystems on small tropical coral islands | Ecological Indicators | | 1470-160X | 2020 | 10.1016/j.ecolind.2020.106656 |
| 53 | Caldera, A; Alvarez-Yépiz, J; Yépez, EA; de los Santos-Villalobos, S | Symbiotic bacteria associated with legume tree species in a Mexican tropical dry forest | Brazilian Journal of Botany | | 0100-8404 | 2023 | 10.1007/s40415-023-00875-y |
| 54 | Carilla, J; Grau, HR | 150 Years of Tree Establishment, Land Use and Climate Change in Montane Grasslands, Northwest Argentina | Biotropica | | 0006-3606 | 2010 | 10.1111/j.1744-7429.2009.00565.x |
| 55 | Ceballos, SJ; Malizia, A | Liana density declined and basal area increased over 12 y in a subtropical montane forest in Argentina | Journal Of Tropical Ecology | | 0266-4674 | 2017 | 10.1017/S0266467417000153 |
| 56 | Chan, N; Takeda, S; Suzuki, R; Yamamoto, S | Establishment of allometric models and estimation of biomass recovery of swidden cultivation fallows in mixed deciduous forests of the Bago Mountains, Myanmar | Forest Ecology and Management | | 0378-1127 | 2013 | 10.1016/j.foreco.2013.05.038 |
| 57 | Chen, J; Feng, K; Hannula, SE; Kuzyakov, Y; Li, YD; Xu, H | Interkingdom plant-microbial ecological networks under selective and clear cutting of tropical rainforest | Forest Ecology and Management | | 0378-1127 | 2021 | 10.1016/j.foreco.2021.119182 |
| 58 | Chernov, TI; Zhelezova, AD; Tkhakakhova, AK; Ksenofontova, NA; Zverev, AO; Tiunov, AV | Soil microbiome, organic matter content and microbial abundance in forest and forest-derived land cover in Cat Tien National Park (Vietnam) | Applied Soil Ecology | | 0929-1393 | 2021 | 10.1016/j.apsoil.2021.103957 |
| 59 | Chikorowondo, G; Muvengwi, J; Mbiba, M; Gandiwa, E | Functional diversity of macroinvertebrates on abandoned cattle enclosures in a semi-arid African savannah | African Journal of Ecology | | 0141-6707 | 2018 | 10.1111/aje.12516 |
| 60 | Chu, Z; Cosset, CCP; Finlayson, C; Cannon, PG; Freckleton, RP; Yusah, KM; Edwards, DP | Tree diversity and liana infestation predict acoustic diversity in logged tropical forests | Biological Conservation | | 0006-3207 | 2024 | 10.1016/j.biocon.2024.110488 |
| 61 | Cinco-Castro, S; Herrera-Silveira, J; Muñoz, JLM; Hernández-Nuñez, H; Hernández, CT | Carbon stock in different ecological types of mangroves in a karstic region (Yucatan, Mexico): an opportunity to avoid site scale emissions | Frontiers In Forests and Global Change | |  | 2023 | 10.3389/ffgc.2023.1181542 |
| 62 | Cole, LES; Bhagwat, SA; Willis, KJ | Fire in the Swamp Forest: Palaeoecological Insights Into Natural and Human-Induced Burning in Intact Tropical Peatlands | Frontiers In Forests and Global Change | |  | 2019 | 10.3389/ffgc.2019.00048 |
| 63 | Cole, RJ; Holl, KD; Keene, CL; Zahawi, RA | Direct seeding of late-successional trees to restore tropical montane forest | Forest Ecology and Management | | 0378-1127 | 2011 | 10.1016/j.foreco.2010.06.038 |
| 64 | Crouzeilles, R; Barros, FSM; Molin, PG; Ferreira, MS; Junqueira, AB; Chazdon, RL; Lindenmayer, DB; Tymus, JRC; Strassburg, BBN; Brancalion, PHS | A new approach to map landscape variation in forest restoration success in tropical and temperate forest biomes | Journal of Applied Ecology | | 0021-8901 | 2019 | 10.1111/1365-2664.13501 |
| 65 | da Silva, KC; Brum, M; Oliveira, RS; Barbosa, BV; Negrao-Rodrigues, V; Teodoro, GS | High resilience of campos rupestres plants to the interaction of drought and fire | Plant Biology | | 1435-8603 | 2023 | 10.1111/plb.13596 |
| 66 | Darusman, T; Murdiyarso, D; Impron, I; Chaniago, IA; Lestari, DP | Carbon Dynamics in Rewetted Tropical Peat Swamp Forests | Climate | |  | 2022 | 10.3390/cli10030035 |
| 67 | de Camargo, PB; Trumbore, SE; Martinelli, LA; Davidson, EA; Nepstad, DC; Victoria, RL | Soil carbon dynamics in regrowing forest of eastern Amazonia | Global Change Biology | | 1354-1013 | 1999 | 10.1046/j.1365-2486.1999.00259.x |
| 68 | Decocq, G; Beina, D; Jamoneau, A; Gourlet-Fleury, S; Closset-Kopp, D | Don't miss the forest for the trees! Evidence for vertical differences in the response of plant diversity to disturbance in a tropical rain forest | Perspectives in Plant Ecology Evolution and Systematics | | 1433-8319 | 2014 | 10.1016/j.ppees.2014.09.001 |
| 69 | Donato, DC; Kauffman, JB; Mackenzie, RA; Ainsworth, A; Pfleeger, AZ | Whole-island carbon stocks in the tropical Pacific: Implications for mangrove conservation and upland restoration | Journal of Environmental Management | | 0301-4797 | 2012 | 10.1016/j.jenvman.2011.12.004 |
| 70 | Dung, LV; Tue, NT; Nhuan, MT; Omori, K | Carbon storage in a restored mangrove forest in Can Gio Mangrove Forest Park, Mekong Delta, Vietnam | Forest Ecology and Management | | 0378-1127 | 2016 | 10.1016/j.foreco.2016.08.032 |
| 71 | Eshel, A; Grünzweig, JM | Root-shoot allometry of tropical forest trees determined in a large-scale aeroponic system | Annals of Botany | | 0305-7364 | 2013 | 10.1093/aob/mcs275 |
| 72 | Ewers, RM; Kliskey, AD; Walker, S; Rutledge, D; Harding, JS; Didham, RK | Past and future trajectories of forest loss in New Zealand | Biological Conservation | | 0006-3207 | 2006 | 10.1016/j.biocon.2006.06.018 |
| 73 | Fagundes, MV; Mazzochini, GG; Ganade, G | The role of plant diversity and facilitation during tropical dry forest restoration | Journal of Ecology | | 0022-0477 | 2023 | 10.1111/1365-2745.14091 |
| 74 | Fauset, S; Gloor, MU; Aidar, MPM; Freitas, HC; Fyllas, NM; Marabesi, MA; Rochelle, AELC; Shenkin, A; Vieira, SA; Joly, CA | Tropical forest light regimes in a human-modified landscape | Ecosphere | | 2150-8925 | 2017 | 10.1002/ecs2.2002 |
| 75 | Feldpausch, TR; Couto, EG; Rodrigues, LC; Pauletto, D; Johnson, MS; Faheyk, TJ; Lehmann, J; Riha, SJ | Nitrogen aboveground turnover and soil stocks to 8 m depth in primary and selectively logged forest in southern Amazonia | Global Change Biology | | 1354-1013 | 2010 | 10.1111/j.1365-2486.2009.02068.x |
| 76 | Ferreira, MC; Cordeiro, AOD; Sampaio, AB; Schmidt, IB; Vieira, DLM | Direct seeding versus seedling planting: survival, biomass, growth, and cost up to 6 years for four tropical seasonal tree species | Restoration Ecology | | 1061-2971 | 2023 | 10.1111/rec.13807 |
| 77 | Franco, AC; Rossatto, DR; Silva, LDR; Ferreira, CD | Cerrado vegetation and global change: the role of functional types, resource availability and disturbance in regulating plant community responses to rising CO2 levels and climate warming | Theoretical and Experimental Plant Physiology | | 2197-0025 | 2014 | 10.1007/s40626-014-0002-6 |
| 78 | Gabey, AM; Gallagher, MW; Whitehead, J; Dorsey, JR; Kaye, PH; Stanley, WR | Measurements and comparison of primary biological aerosol above and below a tropical forest canopy using a dual channel fluorescence spectrometer | Atmospheric Chemistry and Physics | | 1680-7316 | 2010 | 10.5194/acp-10-4453-2010 |
| 79 | Gerlach, MD; Lozano-Baez, SE; Castellini, M; Guzman, N; Gomez, WA; Medina, B | Low Cost and Easy to Implement Physical and Hydrological Soil Assessment of Shade-Grown Coffee in Santa Rosa, Guatemala | Land | |  | 2023 | 10.3390/land12020390 |
| 80 | Giles, AL; Costa, PD; Rowland, L; Abrahao, A; Lobo, L; Verona, L; Silva, MC; Monge, M; Wolfsdorf, G; Petroni, A; D'Angioli, AM; Sampaio, AB; Schimidt, IB; Oliveira, RS | How effective is direct seeding to restore the functional composition of neotropical savannas? | Restoration Ecology | | 1061-2971 | 2022 | 10.1111/rec.13474 |
| 81 | Gomes, ABS; Barros, MF; Vanderlei, RS; Tabarelli, M; Dodonov, P | Plant vegetative propagation plays a considerable role in the regeneration following slash-and-burn agriculture in Caatinga dry forest | Acta Oecologica-International Journal of Ecology | | 1146-609X | 2024 | 10.1016/j.actao.2023.103971 |
| 82 | González-Avila, PA; Luna-Vega, I; Garcia-Sandoval, R; Contreras-Medina, R | Distributional patterns of the Order Gomphales (Fungi: Basidiomycota) in Mexico | Tropical Conservation Science | | 1940-0829 | 2016 | 10.1177/1940082916667140 |
| 83 | González-Rivas, B; Tigabu, M; Castro-Marín, G; Odén, PC | Regeneration of dry forest species Population dynamics and spatial distribution of seedlings and saplings of four dry forest species in Nicaragua | Bois et Forets des Tropiques | | 0006-579X | 2009 |  |
| 84 | Griffiths, LN; Mitsch, WJ | Estimating the Effects of a Hurricane on Carbon Storage in Mangrove Wetlands in Southwest Florida | Plants-Basel | | 2223-7747 | 2021 | 10.3390/plants10081749 |
| 85 | Guedes, BS; Olsson, BA; Sitoe, AA; Egnell, G | Net primary production in plantations of Pinus taeda and Eucalyptus cloeziana compared with a mountain miombo woodland in Mozambique | Global Ecology and Conservation | | 2351-9894 | 2018 | 10.1016/j.gecco.2018.e00414 |
| 86 | Guedje, NM; Zuidema, PA; During, H; Foahom, B; Lejoly, J | Tree bark as a non-timber forest product:: The effect of bark collection on population structure and dynamics of Garcinia lucida Vesque | Forest Ecology and Management | | 0378-1127 | 2007 | 10.1016/j.foreco.2006.09.029 |
| 87 | Gunaratne, AMTA; Gunatilleke, CVS; Gunatilleke, IAUN; Weerasinghe, HMSPM; Burslem, DFRP | Release from root competition promotes tree seedling survival and growth following transplantation into human-induced grasslands in Sri Lanka | Forest Ecology and Management | | 0378-1127 | 2011 | 10.1016/j.foreco.2011.03.027 |
| 88 | Guo, XF; Wang, SJ; Wang, C; Lan, MJ; Yang, SQ; Luo, S; Li, R; Xia, JH; Xiao, B; Xie, LL; Wang, ZJ; Guo, ZP | The Changes, Aggregation Processes, and Driving Factors for Soil Fungal Communities during Tropical Forest Restoration | Journal of Fungi | |  | 2024 | 10.3390/jof10010027 |
| 89 | Gutiérrez-Chacón, C; Dorman, CF; Klein, AM | Forest-edge associated bees benefit from the proportion of tropical forest regardless of its edge length | Biological Conservation | | 0006-3207 | 2018 | 10.1016/j.biocon.2018.02.009 |
| 90 | Harishma, KM; Sandeep, S; Sreekumar, VB | Biomass and carbon stocks in mangrove ecosystems of Kerala, southwest coast of India | Ecological Processes | |  | 2020 | 10.1186/s13717-020-00227-8 |
| 91 | Hasselquist, NJ; Santiago, LS; Allen, MF | Belowground nitrogen dynamics in relation to hurricane damage along a tropical dry forest chronosequence | Biogeochemistry | | 0168-2563 | 2010 | 10.1007/s10533-009-9378-9 |
| 92 | Hattori, D; Kenzo, T; Shirahama, T; Harada, Y; Kendawang, JJ; Ninomiya, I; Sakurai, K | Degradation of soil nutrients and slow recovery of biomass following shifting cultivation in the heath forests of Sarawak, Malaysia | Forest Ecology and Management | | 0378-1127 | 2019 | 10.1016/j.foreco.2018.09.051 |
| 93 | Hieu, PV; Dung, LV; Tue, NT; Omori, K | Will restored mangrove forests enhance sediment organic carbon and ecosystem carbon storage? | Regional Studies in Marine Science | | 2352-4855 | 2017 | 10.1016/j.rsma.2017.05.003 |
| 94 | Hjerpe, J; Hedenås, H; Elmqvist, T | Tropical rain forest recovery from cyclone damage and fire in Samoa | Biotropica | | 0006-3606 | 2001 | 10.1111/j.1744-7429.2001.tb00176.x |
| 95 | Holl, KD | Effects of above- and below-ground competition of shrubs and grass on Calophyllum brasiliense (Camb.) seedling growth in abandoned tropical pasture | Forest Ecology and Management | | 0378-1127 | 1998 | 10.1016/S0378-1127(98)00248-5 |
| 96 | Hooper, E; Condit, R; Legendre, P | Responses of 20 native tree species to reforestation strategies for abandoned farmland in Panama | Ecological Applications | | 1051-0761 | 2002 | 10.2307/3099927 |
| 97 | Houghton, RA; Lawrence, KT; Hackler, JL; Brown, S | The spatial distribution of forest biomass in the Brazilian Amazon: a comparison of estimates | Global Change Biology | | 1354-1013 | 2001 | 10.1046/j.1365-2486.2001.00426.x |
| 98 | Hu, C; Lei, JP; Wan, JZ | The Relationships between Biomass and Soil Respiration across Different Forest Management Practices | Forests | |  | 2024 | 10.3390/f15040712 |
| 99 | Huante, P; Ceccon, E; Orozco-Segovia, A; Sánchez-Coronado, ME; Acosta, I; Rincón, E | THE ROLE OF ARBUSCULAR MYCORRHIZAL FUNGI ON THE EARLY-STAGE RESTORATION OF SEASONALLY DRY TROPICAL FOREST IN CHAMELA, MEXICO | Revista Arvore | | 0100-6762 | 2012 | 10.1590/S0100-67622012000200009 |
| 100 | JANZEN, DH | MANAGEMENT OF HABITAT FRAGMENTS IN A TROPICAL DRY FOREST - GROWTH | Annals of the Missouri Botanical Garden | | 0026-6493 | 1988 | 10.2307/2399468 |
| 101 | Jimenez-Rodriguez, DL; Gao, Y; Solorzano, JV; Skutsch, M; Perez-Salicrup, DR; Salinas-Melgoza, MA; Farfan, M | Mapping Forest Degradation and Contributing Factors in a Tropical Dry Forest | Frontiers In Environmental Science | |  | 2022 | 10.3389/fenvs.2022.912873 |
| 102 | Jin, X; Liu, YJ; Hu, WJ; Wang, GB; Kong, ZY; Wu, L; Ge, G | Soil bacterial and fungal communities and the associated nutrient cycling responses to forest conversion after selective logging in a subtropical forest of China | Forest Ecology And Management | | 0378-1127 | 2019 | 10.1016/j.foreco.2019.04.032 |
| 103 | Johansson, MU; Granström, A | Fire and grazing controlling a tropical tree line: Effects of long-term grazing exclusion in Bale Mountains, Ethiopia | Journal of Vegetation Science | | 1100-9233 | 2020 | 10.1111/jvs.12905 |
| 104 | Jones, FA; Erickson, DL; Bernal, MA; Bermingham, E; Kress, WJ; Herre, EA; Muller-Landau, HC; Turner, BL | The Roots of Diversity: Below Ground Species Richness and Rooting Distributions in a Tropical Forest Revealed by DNA Barcodes and Inverse Modeling | Plos One | | 1932-6203 | 2011 | 10.1371/journal.pone.0024506 |
| 105 | Jones, HP; Nickel, B; Srebotnjak, T; Turner, W; Gonzalez-Roglich, M; Zavaleta, E; Hole, DG | Global hotspots for coastal ecosystem-based adaptation | Plos One | | 1932-6203 | 2020 | 10.1371/journal.pone.0233005 |
| 106 | Kainer, KA; Wadt, LH; Staudhammer, CL | Testing a silvicultural recommendation: Brazil nut responses 10years after liana cutting | Journal of Applied Ecology | | 0021-8901 | 2014 | 10.1111/1365-2664.12231 |
| 107 | Kamo, K; Vacharangkura, T; Tiyanon, S; Viriyabuncha, C; Nimpila, S; Duangsrisen, B; Thaingam, R; Sakai, M | Biomass and dry matter production in planted forests and an adjacent secondary forest in the grassland area of Sakaerat, northeastern Thailand | Tropics | | 0917-415X | 2008 | 10.3759/tropics.17.209 |
| 108 | Karmakar, S; Pradhan, BS; Bhardwaj, A; Pavan, BK; Chaturvedi, R; Chaudhry, P | Assessment of Above- and Below-Ground Carbon Pools in a Tropical Dry Deciduous Forest Ecosystem of Bhopal, India | Chinese Journal of Urban and Environmental Studies | | 2345-7481 | 2020 | 10.1142/S2345748120500219 |
| 109 | Kawahara, M | Ricoh's programs for contributing to betterment of the environment and society | Second International Symposium on Environmentally Conscious Design and Inverse Manufacturing, Proceedings | |  | 2001 |  |
| 110 | Kerdraon, D; Drewer, J; Chung, AYC; Majalap, N; Slade, EM; Bréchet, L; Wallwork, A; Castro-Trujillo, B; Sayer, EJ | Litter Inputs, but Not Litter Diversity, Maintain Soil Processes in Degraded Tropical Forests-A Cross-Continental Comparison | Frontiers in Forests and Global Change | |  | 2020 | 10.3389/ffgc.2019.00090 |
| 111 | Kueffer, C; Schumacher, E; Dietz, H; Fleischmann, K; Edwards, PJ | Managing successional trajectories in alien-dominated, novel ecosystems by facilitating seedling regeneration: A case study | Biological Conservation | | 0006-3207 | 2010 | 10.1016/j.biocon.2010.04.031 |
| 112 | Kumari, S; Maiti, SK | Reclamation of coalmine spoils with topsoil, grass, and legume: a case study from India | Environmental Earth Sciences | | 1866-6280 | 2019 | 10.1007/s12665-019-8446-2 |
| 113 | Lalnunzira, C; Brearley, FQ; Tripathi, SK | Root growth dynamics during recovery of tropical mountain forest in North-east India | Journal of Mountain Science | | 1672-6316 | 2019 | 10.1007/s11629-018-5303-9 |
| 114 | Lefebvre, D; Román-Dañobeytia, F; Soete, J; Cabanillas, F; Corvera, R; Ascorra, C; Fernandez, LE; Silman, M | Biochar Effects on Two Tropical Tree Species and Its Potential as a Tool for Reforestation | Forests | |  | 2019 | 10.3390/f10080678 |
| 115 | Lewis, T; Verstraten, L; Hogg, B; Wehr, BJ; Swift, S; Tindale, N; Menzies, NW; Dalal, RC; Bryant, P; Francis, B; Smith, TE | Reforestation of agricultural land in the tropics: The relative contribution of soil, living biomass and debris pools to carbon sequestration | Science of the Total Environment | | 0048-9697 | 2019 | 10.1016/j.scitotenv.2018.08.351 |
| 116 | Li, HM; Ma, YX; Aide, TM; Liu, WJ | Past, present and future land-use in Xishuangbanna, China and the implications for carbon dynamics | Forest Ecology and Management | | 0378-1127 | 2008 | 10.1016/j.foreco.2007.06.051 |
| 117 | Liu, LB; Wu, YY; Hu, G; Zhang, ZH; Cheng, AY; Wang, SJ; Ni, J | Biomass of karst evergreen and deciduous broad-leaved mixed forest in central Guizhou province, southwestern China: a comprehensive inventory of a 2 ha plot | Silva Fennica | | 0037-5330 | 2016 | 10.14214/sf.1492 |
| 118 | Long, MS; Litton, CM; Giardina, CP; Deenik, J; Cole, RJ; Sparks, JP | Impact of nonnative feral pig removal on soil structure and nutrient availability in Hawaiian tropical montane wet forests | Biological Invasions | | 1387-3547 | 2017 | 10.1007/s10530-017-1368-6 |
| 119 | Luo, YQ; Zhao, XY; Li, YQ; Liu, XP; Wang, LL; Wang, XY; Du, Z | Wind disturbance on litter production affects soil carbon accumulation in degraded sandy grasslands in semi-arid sandy grassland | Ecological Engineering | | 0925-8574 | 2021 | 10.1016/j.ecoleng.2021.106373 |
| 120 | Mackey, B; Kormos, CF; Keith, H; Moomaw, WR; Houghton, RA; Mittermeier, RA; Hole, D; Hugh, S | Understanding the importance of primary tropical forest protection as a mitigation strategy | Mitigation and Adaptation Strategies for Global Change | | 1381-2386 | 2020 | 10.1007/s11027-019-09891-4 |
| 121 | Maltz, MR; Treseder, KK | Sources of inocula influence mycorrhizal colonization of plants in restoration projects: a meta-analysis | Restoration Ecology | | 1061-2971 | 2015 | 10.1111/rec.12231 |
| 122 | Mande, HK; Abdullah, AM; Aris, AZ; Ainuddin, AN | Factors responsible for spatial and temporal variation of soil CO2 efflux in a 50 year recovering tropical forest, Peninsular Malaysia | Environmental Earth Sciences | | 1866-6280 | 2015 | 10.1007/s12665-014-3810-8 |
| 123 | Mande, HK; Abdullah, AM; Aris, AZ; Nuruddin, AA | A Comparison of Soil CO2 Efflux Rate in Young Rubber Plantation, Oil Palm Plantation, Recovering and Primary Forest Ecosystems of Malaysia | Polish Journal of Environmental Studies | | 1230-1485 | 2014 |  |
| 124 | Marshall, A; McLaughlin, BP; Zerr, C; Yanguas-Fernández, E; Hall, JS | Early indications of success rehabilitating an underperforming teak (Tectona grandis) plantation in Panama through enrichment planting | New Forests | | 0169-4286 | 2021 | 10.1007/s11056-020-09801-6 |
| 125 | Matheny, AM; Garrity, SR; Bohrer, G | The Calibration and Use of Capacitance Sensors to Monitor Stem Water Content in Trees | Jove-Journal of Visualized Experiments | | 1940-087X | 2017 | 10.3791/57062 |
| 126 | Melone, A; Bremer, LL; Crow, SE; Hastings, Z; Winter, KB; Ticktin, T; Rii, YM; Wong, ML; Kukea-Shultz, K; Watson, SJ; Trauernicht, C | Assessing Baseline Carbon Stocks for Forest Transitions: A Case Study of Agroforestry Restoration from Hawai'i | Agriculture-Basel | |  | 2021 | 10.3390/agriculture11030189 |
| 127 | Mishra, S; Page, SE; Cobb, AR; Lee, JSH; Jovani-Sancho, AJ; Sjögersten, S; Jaya, A; Aswandi; Wardle, DA | Degradation of Southeast Asian tropical peatlands and integrated strategies for their better management and restoration | Journal of Applied Ecology | | 0021-8901 | 2021 | 10.1111/1365-2664.13905 |
| 128 | Mitchard, ETA | The tropical forest carbon cycle and climate change | Nature | | 0028-0836 | 2018 | 10.1038/s41586-018-0300-2 |
| 129 | Miyamoto, K; Aiba, SI; Aoyagi, R; Nilus, R | Logging impacts on above- and belowground forest biomass and production in Bornean lowland forests | Tropics | | 0917-415X | 2024 | 10.3759/tropics.MS23-09 |
| 130 | Montiel-González, C; Bravo-Monzón, AE; Flores-Puerto, JI; Valadez-Cortés, F; Azcoytia-Escalona, LE; García-Oliva, F; Arena-Ortiz, ML; Alvarez-Añorve, MY; Avila-Cabadilla, LD | Disturbance Level Mediates the Differential Resistance of Tropical Dry Forest Soil and Vegetation Attributes to High-Intensity Hurricanes | Ecosystems | | 1432-9840 | 2024 | 10.1007/s10021-024-00905-0 |
| 131 | Moore, J; Macalady, JL; Schulz, MS; White, AF; Brantley, SL | Shifting microbial community structure across a marine terrace grassland chronosequence, Santa Cruz, California | Soil Biology & Biochemistry | | 0038-0717 | 2010 | 10.1016/j.soilbio.2009.09.015 |
| 132 | Nakagawa, M; Momose, K; Kishimoto-Yamada, K; Kamoi, T; Tanaka, HO; Kaga, M; Yamashita, S; Itioka, T; Nagamasu, H; Sakai, S; Nakashizuka, T | Tree community structure, dynamics, and diversity partitioning in a Bornean tropical forested landscape | Biodiversity And Conservation | | 0960-3115 | 2013 | 10.1007/s10531-012-0405-0 |
| 133 | Narayana, J; Shashidhar; Nanda, A; Savinaya, MS | Carbon Sequestration Potential of Trees in Kuvempu University Campus Forest Area, Western Ghats, Karnataka | Socio-Economic And Eco-Biological Dimensions in Resource Use And Conservation: Strategies for Sustainability | | 1431-6250 | 2020 | 10.1007/978-3-030-32463-6_15 |
| 134 | Needham, JF; Arellano, G; Davies, SJ; Fisher, RA; Hammer, V; Knox, RG; Mitre, D; Muller-Landau, HC; Zuleta, D; Koven, CD | Tree crown damage and its effects on forest carbon cycling in a tropical forest | Global Change Biology | | 1354-1013 | 2022 | 10.1111/gcb.16318 |
| 135 | Nogueira, LR; Engel, VL; Parrotta, JA; de Melo, ACG; Ré, DS | Allometric equations for estimating tree biomass in restored mixed-species Atlantic Forest stands | Biota Neotropica | | 1676-0603 | 2014 | 10.1590/1676-06032013008413 |
| 136 | Ouaadi, N; Jarlan, L; Villard, L; Chakir, A; Khabba, S; Fanise, P; Kasbani, M; Rafi, Z; Le Dantec, V; Ezzahar, J; Frison, PL | Temporal decorrelation of C-band radar data over wheat in a semi-arid area using sub-daily tower-based observations | Remote Sensing of Environment | | 0034-4257 | 2024 | 10.1016/j.rse.2024.114059 |
| 137 | Pati, PK; Kaushik, P; Khan, ML; Khare, PK | Allometric equations for biomass and carbon stock estimation of small diameter woody species from tropical dry deciduous forests: Support to REDD+ | Trees Forests and People | |  | 2022 | 10.1016/j.tfp.2022.100289 |
| 138 | Pauw, A; Van Bael, SA; Peters, HA; Allison, SD; Camargo, JLC; Cifuentes-Jara, M; Conserva, A; Restom, TG; Heartsill-Scalley, T; Mangan, SA; Nunez-Iturri, G; Rivera-Ocasio, E; Rountree, M; Vetter, S; de Castilho, CV | Physical damage in relation to carbon allocation strategies of tropical forest tree saplings | Biotropica | | 0006-3606 | 2004 |  |
| 139 | Phillips, ML; Aronson, EL; Maltz, MR; Allen, EB | Native and invasive inoculation sources modify fungal community assembly and biomass production of a chaparral shrub | Applied Soil Ecology | | 0929-1393 | 2020 | 10.1016/j.apsoil.2019.103370 |
| 140 | Preece, ND; Lawes, MJ; Rossman, AK; Curran, TJ; van Oosterzee, P | Modelling the growth of young rainforest trees for biomass estimates and carbon sequestration accounting | Forest Ecology and Management | | 0378-1127 | 2015 | 10.1016/j.foreco.2015.05.003 |
| 141 | Proctor, S; McClean, CJ; Hill, JK | Protected areas of Borneo fail to protect forest landscapes with high habitat connectivity | Biodiversity and Conservation | | 0960-3115 | 2011 | 10.1007/s10531-011-0099-8 |
| 142 | Purnomo, DW; Prasetyo, LB; Widyatmoko, D; Rushayati, SB; Usmadi, D; Zulkarnaen, RN; Yudaputra, A; Cropper, W | Potential species for high biomass production and allometric modelling of even-aged native tropical lowland trees of Indonesia | Acta Botanica Brasilica | | 0102-3306 | 2024 | 10.1590/1677-941X-ABB-2023-0073 |
| 143 | Radabaugh, KR; Moyer, RP; Chappel, AR; Dontis, EE; Russo, CE; Joyse, KM; Bownik, MW; Goeckner, AH; Khan, NS | Mangrove Damage, Delayed Mortality, and Early Recovery Following Hurricane Irma at Two Landfall Sites in Southwest Florida, USA | Estuaries and Coasts | | 1559-2723 | 2020 | 10.1007/s12237-019-00564-8 |
| 144 | Ray, R; Chowdhury, C; Majumder, N; Dutta, MK; Mukhopadhyay, SK; Jana, TK | Improved model calculation of atmospheric CO2 increment in affecting carbon stock of tropical mangrove forest | Tellus Series B-Chemical and Physical Meteorology | | 1600-0889 | 2013 | 10.3402/tellusb.v65i0.18981 |
| 145 | Ray, R; Ganguly, D; Chowdhury, C; Dey, M; Das, S; Dutta, MK; Mandal, SK; Majumder, N; De, TK; Mukhopadhyay, SK; Jana, TK | Carbon sequestration and annual increase of carbon stock in a mangrove forest | Atmospheric Environment | | 1352-2310 | 2011 | 10.1016/j.atmosenv.2011.04.074 |
| 146 | Rehm, EM; Yelenik, SG; Smith, MP; D'Antonio, CM | Architecture of remnant trees influences native woody plant recruitment in abandoned Hawaiian pastures | Plant Ecology | | 1385-0237 | 2021 | 10.1007/s11258-020-01072-7 |
| 147 | Rivera-Ocasio, E; Aide, TM; Rios-López, N | The effects of salinity on the dynamics of a Pterocarpus officinalis forest stand in Puerto Rico | Journal of Tropical Ecology | | 0266-4674 | 2007 | 10.1017/S0266467407004361 |
| 148 | Robinson, SJB; Elias, D; Johnson, D; Both, S; Riutta, T; Goodall, T; Majalap, N; McNamara, NP; Griffiths, R; Ostle, N | Soil Fungal Community Characteristics and Mycelial Production Across a Disturbance Gradient in Lowland Dipterocarp Rainforest in Borneo | Frontiers In Forests and Global Change | |  | 2020 | 10.3389/ffgc.2020.00064 |
| 149 | Rodríguez-León, CH; Roa-Fuentes, LL; Sterling, A; Suárez, JC | Plant Biodiversity Homogenization across the Chronosequence in Highly Fragmented Landscapes in the Colombian Andean-Amazonian Transition | Forests | |  | 2022 | 10.3390/f13091422 |
| 150 | Ruiz, DEM; Aryal, DR; Ruiz, RP; Hernández, FG; Lugo, FC; López, GV | Carbon contents and fine root production in tropical silvopastoral systems | Land Degradation & Development | | 1085-3278 | 2021 | 10.1002/ldr.3761 |
| 151 | Saner, P; Loh, YY; Ong, RC; Hector, A | Carbon Stocks and Fluxes in Tropical Lowland Dipterocarp Rainforests in Sabah, Malaysian Borneo | Plos One | | 1932-6203 | 2012 | 10.1371/journal.pone.0029642 |
| 152 | Saragi-Sasmito, MF; Murdiyarso, D; June, T; Sasmito, SD | Carbon stocks, emissions, and aboveground productivity in restored secondary tropical peat swamp forests | Mitigation and Adaptation Strategies for Global Change | | 1381-2386 | 2019 | 10.1007/s11027-018-9793-0 |
| 153 | Scheer, MB | Mineral nutrient fluxes in rainfall and throughfall in a lowland Atlantic rainforest in southern Brazil | Journal of Forest Research | | 1341-6979 | 2011 | 10.1007/s10310-010-0222-9 |
| 154 | Schwilk, DW; Gaetani, MS; Poulos, HM | Oak Bark Allometry and Fire Survival Strategies in the Chihuahuan Desert Sky Islands, Texas, USA | Plos One | | 1932-6203 | 2013 | 10.1371/journal.pone.0079285 |
| 155 | Scowcroft, PG; Meinzer, FC; Goldstein, G; Melcher, PJ; Jeffrey, J | Moderating night radiative cooling reduces frost damage to Metrosideros polymorpha seedlings used for forest restoration in Hawaii | Restoration Ecology | | 1061-2971 | 2000 | 10.1046/j.1526-100x.2000.80023.x |
| 156 | Shi, LL; Feng, WT; Xu, JC; Kuzyakov, Y | Agroforestry systems: Meta-analysis of soil carbon stocks, sequestration processes, and future potentials | Land Degradation & Development | | 1085-3278 | 2018 | 10.1002/ldr.3136 |
| 157 | Silva, CVJ; Aragao, LEOC; Barlow, J; Espirito-Santo, F; Young, PJ; Anderson, LO; Berenguer, E; Brasil, I; Brown, IF; Castro, B; Farias, R; Ferreira, J; França, F; Graça, PMLA; Kirsten, L; Lopes, AP; Salimon, C; Scaranello, MA; Seixas, M; Souza, FC; Xaud, HAM | Drought-induced Amazonian wildfires instigate a decadal-scale disruption of forest carbon dynamics | Philosophical Transactions of The Royal Society B-Biological Sciences | | 0962-8436 | 2018 | 10.1098/rstb.2018.0043 |
| 158 | Silver, WL; Ostertag, R; Lugo, AE | The potential for carbon sequestration through reforestation of abandoned tropical agricultural and pasture lands | Restoration Ecology | | 1061-2971 | 2000 | 10.1046/j.1526-100x.2000.80054.x |
| 159 | Silver, WL; Scatena, FN; Johnson, AH; Siccama, TG; Watt, F | At what temporal scales does disturbance affect belowground nutrient pools? | Biotropica | | 0006-3606 | 1996 | 10.2307/2389087 |
| 160 | Sinclair, AL; Graham, LLB; Putra, EI; Saharjo, BH; Applegate, G; Grover, SP; Cochrane, MA | Effects of distance from canal and degradation history on peat bulk density in a degraded tropical peatland | Science of the Total Environment | | 0048-9697 | 2020 | 10.1016/j.scitotenv.2019.134199 |
| 161 | Singh, AN; Singh, JS | Biomass, net primary production and impact of bamboo plantation on soil redevelopment in a dry tropical region | Forest Ecology and Management | | 0378-1127 | 1999 | 10.1016/S0378-1127(98)00523-4 |
| 162 | Singh, D; Slik, JWF; Jeon, YS; Tomlinson, KW; Yang, XD; Wang, J; Kerfahi, D; Porazinska, DL; Adams, JM | Tropical forest conversion to rubber plantation affects soil micro- & mesofaunal community & diversity | Scientific Reports | | 2045-2322 | 2019 | 10.1038/s41598-019-42333-4 |
| 163 | Singh, RS; Tripathi, N; Chaulya, SK | Ecological study of revegetated coal mine spoil of an Indian dry tropical ecosystem along an age gradient | Biodegradation | | 0923-9820 | 2012 | 10.1007/s10532-012-9573-6 |
| 164 | Smith, AP; Marín-Spiotta, E; Balser, T | Successional and seasonal variations in soil and litter microbial community structure and function during tropical postagricultural forest regeneration: a multiyear study | Global Change Biology | | 1354-1013 | 2015 | 10.1111/gcb.12947 |
| 165 | Soto-Navarro, C; Ravilious, C; Arnell, A; de Lamo, X; Harfoot, M; Hill, SLL; Wearn, OR; Santoro, M; Bouvet, A; Mermoz, S; Toan, TL; Xia, J; Liu, S; Yuan, W; Spawn, SA; Gibbs, HK; Ferrier, S; Harwood, T; Alkemade, R; Schipper, AM; Schmidt-Traub, G; Strassburg, B; Miles, L; Burgess, ND; Kapos, V | Mapping co-benefits for carbon storage and biodiversity to inform conservation policy and action | Philosophical Transactions of The Royal Society B-Biological Sciences | | 0962-8436 | 2020 | 10.1098/rstb.2019.0128 |
| 166 | Sultana, F; Arfin-Khan, MAS; Karim, MR; Mukul, SA | Rainfall Modifies the Disturbance Effects on Regulating Ecosystem Services in Tropical Forests of Bangladesh | Forests | |  | 2023 | 10.3390/f14020272 |
| 167 | Teh, YA; Silver, WL; Scatena, FN | A decade of belowground reorganization following multiple disturbances in a subtropical wet forest | Plant and Soil | | 0032-079X | 2009 | 10.1007/s11104-009-9926-z |
| 168 | Torres, JR; Sanchez-Mejia, ZM; Alcudia-Aguilar, A; Medrano-Pérez, OR; Barraza-Guardado, RH; Suzuky-Pinto, R | Estimation of Mangrove Blue Carbon in Three Semi-arid Lagoons in the Gulf of California | Wetlands | | 0277-5212 | 2023 | 10.1007/s13157-023-01659-6 |
| 169 | Traoré, S; Zo-Bi, IC; Piponiot, C; Aussenac, R; Hérault, B | Fragmentation is the main driver of residual forest aboveground biomass in West African low forest-high deforestation landscapes | Trees Forests and People | |  | 2024 | 10.1016/j.tfp.2023.100477 |
| 170 | Tripathi, N; Singh, RS | Ecological restoration of mined-out areas of dry tropical environment, India | Environmental Monitoring and Assessment | | 0167-6369 | 2008 | 10.1007/s10661-007-0083-7 |
| 171 | Tripathi, N; Singh, RS; Nathanail, CP | Mine spoil acts as a sink of carbon dioxide in Indian dry tropical environment | Science of the Total Environment | | 0048-9697 | 2014 | 10.1016/j.scitotenv.2013.09.024 |
| 172 | Vaidyanathan, S; Krishnaswamy, J; Kumar, NS; Dhanwatey, H; Dhanwatey, P; Karanth, KU | Patterns of tropical forest dynamics and human impacts: Views from above and below the canopy | Biological Conservation | | 0006-3207 | 2010 | 10.1016/j.biocon.2010.04.027 |
| 173 | Vargas, R; Allen, EB; Allen, MF | Effects of Vegetation Thinning on Above- and Belowground Carbon in a Seasonally Dry Tropical Forest in Mexico | Biotropica | | 0006-3606 | 2009 | 10.1111/j.1744-7429.2009.00494.x |
| 174 | Vargas, R; Allen, MF; Allen, EB | Biomass and carbon accumulation in a fire chronosequence of a seasonally dry tropical forest | Global Change Biology | | 1354-1013 | 2008 | 10.1111/j.1365-2486.2007.01462.x |
| 175 | Vargas, R; Hasselquist, N; Allen, EB; Allen, MF | Effects of a Hurricane Disturbance on Aboveground Forest Structure, Arbuscular Mycorrhizae and Belowground Carbon in a Restored Tropical Forest | Ecosystems | | 1432-9840 | 2010 | 10.1007/s10021-009-9305-x |
| 176 | Vargas, R; Trumbore, SE; Allen, MF | Evidence of old carbon used to grow new fine roots in a tropical forest | New Phytologist | | 0028-646X | 2009 | 10.1111/j.1469-8137.2009.02789.x |
| 177 | Varma, V; Catherin, AM; Sankaran, M | Effects of increased N and P availability on biomass allocation and root carbohydrate reserves differ between N-fixing and non-N-fixing savanna tree seedlings | Ecology and Evolution | | 2045-7758 | 2018 | 10.1002/ece3.4289 |
| 178 | Vasagadekar, PR; Gargate, AV; Patil, YY; Raut, PD | Carbon sequestration potential of trees from urban green spaces of Kolhapur city, Maharashtra, India | Environmental & Socio-Economic Studies | | 2354-0079 | 2023 | 10.2478/environ-2023-0014 |
| 179 | Veintimilla, RAR; MacFarlane, D; Cooper, L | The carbon sequestration potential of 'analog' forestry in Ecuador: an alternative strategy for reforestation of degraded pastures | Forestry | | 0015-752X | 2021 | 10.1093/forestry/cpaa017 |
| 180 | Vila, LM; Ménager, M; Finegan, B; Delgado, D; Casanoves, F; Salas, LAA; Castillo, M; Sánchez, LGH; Méndez, Y; Toruño, HS; Solano, G; Mora, PZ; Bieng, MAN | Above-ground biomass storage potential in primary rain forests managed for timber production in Costa Rica | Forest Ecology and Management | | 0378-1127 | 2021 | 10.1016/j.foreco.2021.119462 |
| 181 | Wang, SJ; Zhang, KF; Fan, YX; Zhang, LL; Guo, XF; Xie, LL; Xiao, B; Wang, ZJ; Guo, ZP | Linkages between bacteria and nutrient availabilities in slash-and-burn tropical soils vary with feeding-habit ants | Land Degradation & Development | | 1085-3278 | 2023 | 10.1002/ldr.4724 |
| 182 | Wang, SJ; Zhao, S; Yang, B; Zhang, KF; Fan, YX; Zhang, LL; Yang, XD | The carbon and nitrogen stoichiometry in litter-soil-microbe continuum rather than plant diversity primarily shapes the changes in bacterial communities along a tropical forest restoration chronosequence | Catena | | 0341-8162 | 2022 | 10.1016/j.catena.2022.106202 |
| 183 | Wantzen, KM | Physical pollution: effects of gully erosion on benthic invertebrates in a tropical clear-water stream | Aquatic Conservation-Marine and Freshwater Ecosystems | | 1052-7613 | 2006 | 10.1002/aqc.813 |
| 184 | Wei, XH; Li, QL; Liu, YQ; Liu, SR; Guo, XM; Zhang, L; Niu, DK; Zhang, WY | Restoring ecosystem carbon sequestration through afforestation: A sub-tropic restoration case study | Forest Ecology and Management | | 0378-1127 | 2013 | 10.1016/j.foreco.2012.06.018 |
| 185 | Werden, LK; Averill, C; Crowther, TW; Calderón-Morales, E; Toro, L; Alvarado, JP; Gutiérrez, LM; Mallory, DE; Powers, JS | Below-ground traits mediate tree survival in a tropical dry forest restoration | Philosophical Transactions of The Royal Society B-Biological Sciences | | 0962-8436 | 2023 | 10.1098/rstb.2021.0067 |
| 186 | Wijas, B; Atkinson, J | Termites in restoration: the forgotten insect? | Restoration Ecology | | 1061-2971 | 2021 | 10.1111/rec.13511 |
| 187 | Williams-Linera, G; Bonilla-Moheno, M; López-Barrera, F | Tropical cloud forest recovery: the role of seed banks in pastures dominated by an exotic grass | New Forests | | 0169-4286 | 2016 | 10.1007/s11056-016-9526-8 |
| 188 | Wösten, JHM; Van der Berg, J; Van Eijk, P; Gevers, GJM; Giesen, WBJT; Hooijer, A; Idris, A; Leenman, PH; Rais, DS; Siderius, C; Silvius, MJ; Suryadiputra, N; Wibisono, IT | Interrelationships between hydrology and ecology in fire degraded tropical peat swamp forests | International Journal of Water Resources Development | | 0790-0627 | 2006 | 10.1080/07900620500405973 |
| 189 | Xanthopoulos, G; Radoglou, K; Derrien, D; Spyroglou, G; Angeli, N; Tsioni, G; Fotelli, MN | Carbon sequestration and soil nitrogen enrichment in Robinia pseudoacacia L. post-mining restoration plantations | Frontiers in Forests and Global Change | |  | 2023 | 10.3389/ffgc.2023.1190026 |
| 190 | Yu, JJ; Cong, W; Ding, Y; Jin, LX; Cong, J; Zhang, YG | Interkingdom Plant-Soil Microbial Ecological Network Analysis under Different Anthropogenic Impacts in a Tropical Rainforest | Forests | |  | 2022 | 10.3390/f13081167 |
| 191 | Yu, Z; Liang, KN; Huang, GH; Wang, XB; Lin, MP; Chen, YL; Zhou, ZZ | Soil Bacterial Community Shifts Are Driven by Soil Nutrient Availability along a Teak Plantation Chronosequence in Tropical Forests in China | Biology-Basel | |  | 2021 | 10.3390/biology10121329 |
| 192 | Zahawi, RA; Holl, KD | Comparing the Performance of Tree Stakes and Seedlings to Restore Abandoned Tropical Pastures | Restoration Ecology | | 1061-2971 | 2009 | 10.1111/j.1526-100X.2008.00423.x |
| 193 | Zanini, AM; Mayrinck, RC; Vieira, SA; de Camargo, PB; Rodrigues, RR | The effect of ecological restoration methods on carbon stocks in the Brazilian Atlantic Forest | Forest Ecology and Management | | 0378-1127 | 2021 | 10.1016/j.foreco.2020.118734 |
| 194 | ZARIN, DJ; JOHNSON, AH | NUTRIENT ACCUMULATION DURING PRIMARY SUCCESSION IN A MONTANE TROPICAL FOREST, PUERTO-RICO | Soil Science Society of America Journal | | 0361-5995 | 1995 | 10.2136/sssaj1995.03615995005900050034x |
| 195 | Zhang, H; Li, YS; Xu, YH; John, R | The recovery of soil N-cycling and P-cycling following reforestation in a degraded tropical limestone mine | Journal of Cleaner Production | | 0959-6526 | 2024 | 10.1016/j.jclepro.2024.141580 |
| 196 | Zhang, MX; Fellowes, JR; Jiang, XL; Wang, W; Chan, BPL; Ren, GP; Zhu, JG | Degradation of tropical forest in Hainan, China, 1991-2008: Conservation implications for Hainan Gibbon (Nomascus hainanus) | Biological Conservation | | 0006-3207 | 2010 | 10.1016/j.biocon.2010.03.014 |
